# SenExo‐cCCT2 Reprograms Senescence Response and Anti‐Tumor Immunity Following FOLFIRINOX Chemotherapy in Pancreatic Ductal Adenocarcinoma

**DOI:** 10.1002/advs.202508431

**Published:** 2025-07-21

**Authors:** Shuncang Zhu, Yinhao Chen, Hongyi Lin, Jinpeng Lu, Yu Pan, Ge Li, Yongding Wu, Haoxiang Zhang, Xiaoxiao Huang, Yiting Chen, Huangjing Chen, Long Jin, Min Huang, Chengyu Liao, Long Huang, Yifeng Tian, Zuwei Wang, Qiaowei Li, Shi Chen

**Affiliations:** ^1^ Shengli Clinical Medical College of Fujian Medical University Fuzhou 350001 P. R. China; ^2^ Department of Hepatobiliary Pancreatic Surgery Fuzhou University Affiliated Provincial Hospital Fujian Provincial Hospital, Fuzhou University Fuzhou 350001 P. R. China; ^3^ Department of General Surgery Fujian Medical University Union Hospital Fuzhou 350001 P. R. China; ^4^ Department of Hepatobiliary Surgery Fujian Institute of Hepatobiliary Surgery Fujian Medical University Union Hospital Fuzhou 350001 P. R. China; ^5^ Fuzhou University Fuzhou 350001 P. R. China; ^6^ College of Biological Science and Engineering Fuzhou University Fuzhou 350108 P. R. China; ^7^ Institute of Applied Genomics Fuzhou University Fuzhou 350108 P. R. China; ^8^ Department of Pathology Fuzhou University Affiliated Provincial Hospital Fujian Provincial Hospital Fuzhou 350001 P. R. China; ^9^ Fujian Provincial Institute of Clinical Geriatrics Fuzhou 350001 P. R. China; ^10^ Fujian Key Laboratory of Geriatrics Fuzhou 350001 P. R. China; ^11^ Fujian Provincial Center for Geriatrics Fuzhou 350001 P. R. China

**Keywords:** CD8^+^ T‐cells, circRNA, chemotherapy, pancreatic ductal adenocarcinoma, senescence

## Abstract

Combination chemotherapy and immunotherapy have failed to achieve breakthroughs in pancreatic ductal adenocarcinoma (PDAC). Chemotherapy‐induced senescence is a potential solution for this problem. This study integrates clinical samples with single‐cell transcriptomic sequencing, proteomics, and RNA sequencing and reveals that FOLFIRINOX (a combination regimen of 5‐fluorouracil, oxaliplatin, irinotecan, and leucovorin) treatment induces a higher proportion of senescent tumor cells (senTCs). This phenomenon is principally attributed to the presence of cCCT2, which inhibits SLX4 condensate‐mediated DNA damage repair pathways by regulating small ubiquitin‐like modifier conjugation, thereby promoting tumor cell senescence. In the tumor immune microenvironment, cCCT2‐overexpressing senTCs exhibit a senescence‐associated secretory phenotype (SASP) with preferential secretion of CXCL10, which induces chemotaxis of CD8^+^ T‐cells. Based on the pro‐senescence and immune‐microenvironment‐remodeling effects of cCCT2, an engineered exosome‐loaded circRNA system, SenExo‐cCCT2 is developed. When combined with SenExo‐cCCT2, the FOLFIRINOX regimen enhances the capacity of pancreatic cancer cells to induce senescence. Subsequently, anti‐PD‐L1 therapy facilitates the immune‐mediated clearance of senTCs, markedly improving the therapeutic efficacy of combined chemotherapy and immunotherapy for pancreatic cancer.

## Graphical abstract

This study demonstrates that SenExo‐cCCT2 enables targeted delivery of cCCT2 to pancreatic cancer cells, where it disrupts IPO13‐mediated nuclear translocation of UBC9, impairing SUMOylation‐dependent DNA damage repair and inducing senescence. Senescent tumor cells secrete CXCL10 to recruit CD8⁺ T‐cells while upregulating PD‐L1. This dual mechanism enhances chemosensitivity and sets the stage for effective sequential immunotherapy.

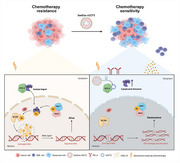

## 1 Introduction

Pancreatic ductal adenocarcinoma (PDAC) is highly malignant, and chemotherapy is the first‐line treatment for progressive PDAC.^[^ ^1^, ^2^ ^]^ The main treatment regimens for this disease include FOLFIRINOX (a combination regimen of 5‐fluorouracil, oxaliplatin, irinotecan, and leucovorin) and AG (gemcitabine plus nab‐paclitaxel). Although FOLFIRINOX exhibits better therapeutic efficacy than AG in clinical treatment,^[^ ^3^, ^4^ ^]^ drug resistance remains a major challenge.^[^ ^5^, ^6^, ^7^ ^]^ Therefore, elucidation of the molecular mechanisms underlying chemoresistance and identification of strategies for enhancing chemosensitivity are urgently required. Some research groups have attempted to increase sensitivity to chemotherapy by combining chemotherapy and immunotherapy, but their clinical efficacy has been limited.^[^ ^8^, ^9^, ^10^ ^]^ These limitations are related to the heterogeneity of the immune microenvironment in PDAC, including variable degrees of CD8^+^ T‐cell infiltration and differential programmed death‐ligand 1 (PD‐L1) expression in tumor cells.^[^ ^11^, ^12^ ^]^ PD‐L1 expression remains an important marker of immunotherapy sensitivity in patients with PDAC but tends to be underexpressed in these patients.^[^ ^13^, ^14^ ^]^ Thus, there is a critical need to discover additional mechanisms that determine the treatment response.

Current chemotherapy‐related research predominantly focuses on drug‐induced apoptosis,^[^ ^15^ ^]^ paradoxically neglecting the critical role of therapy‐induced cellular senescence. We observed that compared with the AG regimen, FOLFIRINOX induced more pronounced senescent phenotypes in pancreatic cancer cells, and the degree of senescence was positively correlated with chemotherapy response and prognosis. Senescent tumor cells (senTCs) promote the development of the senescence‐associated secretory phenotype (SASP) in the tumor microenvironment, which recruits CD8^+^ T‐cells to infiltrate the tumor environment from the peripheral circulation.^[^ ^16^, ^17^, ^18^ ^]^ However, senescence is a double‐edged sword. Although chemotherapy‐induced tumor cell senescence initially serves as an indicator of chemosensitivity, persistent senTCs can escape growth arrest after exiting dormancy during tumor microenvironment remodeling, resulting in increased stemness and drug resistance.^[^ ^19^, ^20^ ^]^ Moreover, senTCs tend to exhibit increased expression of the immune checkpoint cosuppression molecule PD‐L1, thereby evading immune‐mediated clearance.^[^ ^21^, ^22^ ^]^ The combination of anti‐PD‐L1 therapy and senescent immune clearance strategies appears to be a feasible and potentially synergistic strategy to increase chemotherapy sensitivity.

Enhancing the induction of senTCs following chemotherapy is a potential strategy to circumvent therapeutic limitations. Current approaches for promoting senescence predominantly rely on amplifying nuclear DNA damage to trigger irreversible cell cycle arrest, with chemotherapeutic agents serving as the principal modality.^[^ ^23^, ^24^ ^]^ The intrinsic capacity for DNA damage repair (DDR) remains a critical determinant of chemoresistance.^[^ ^25^ ^]^ FOLFIRINOX, a powerful chemotherapy regimen consisting of a four‐drug combination, induces various types of DNA damage, including DNA‐protein crosslinks (DPCs), inter‐strand crosslinks (ICLs), and double‐strand breaks (DSBs).^[^ ^3^, ^5^ ^]^ Consequently, the FOLFIRINOX regimen increased the senescent cell burden. However, although patients initially treated with FOLFIRINOX exhibit enhanced sensitivity, they may subsequently develop resistance and relapse owing to an immunosuppressive tumor microenvironment. SLX4 operates as a scaffold protein within repair complexes, orchestrating phase‐separation‐driven biomolecular condensates, which play a crucial role in various forms of DDR.^[^ ^26^, ^27^ ^]^ Therefore, SLX4 likely mediates FOLFIRINOX‐induced tumor cell senescence. Unraveling the molecular mechanisms governing the formation of SLX4 condensates holds promise for enhancing the chemosensitivity of cells to FOLFIRINOX.

Accumulating evidence has implicated circular RNAs (circRNAs) as regulators of DDR pathways. Previous studies have demonstrated that circRNAs not only mediate chemoresistance but also modulate cellular senescence and immune cell infiltration.^[^ ^28^, ^29^, ^30^, ^31^, ^32^ ^]^ Therefore, we hypothesized that circRNAs enhance chemosensitivity by orchestrating DDR pathways to drive senescence and immune microenvironment remodeling. In this study, we identified cCCT2, a senescence‐associated circRNA upregulated in chemotherapy‐responsive PDAC patients, which enhances tumor cell senescence by impairing the small ​ubiquitin‐like modifier conjugation (SUMOylation) of SLX4. Using in vitro and in vivo models, we demonstrated that cCCT2 enhanced chemosensitization and promoted immune infiltration via senescence potentiation. Therefore, we engineered a biocompatible exosome‐based delivery platform to achieve efficient circRNA therapeutic payload delivery, SenExo‐cCCT2. SenExo‐cCCT2 combined with FOLFIRINOX chemotherapy‐induced senescence and sequential treatment with an anti‐PD‐L1 antibody effectively removed senTCs, increasing the therapeutic effect of chemotherapy in PDAC.

## 2 Results

### 2.1 Correlation of Tumor Cell Senescence with Chemosensitivity and CD8^+^ T‐Cell Infiltration in PDAC

**Figure 1:**
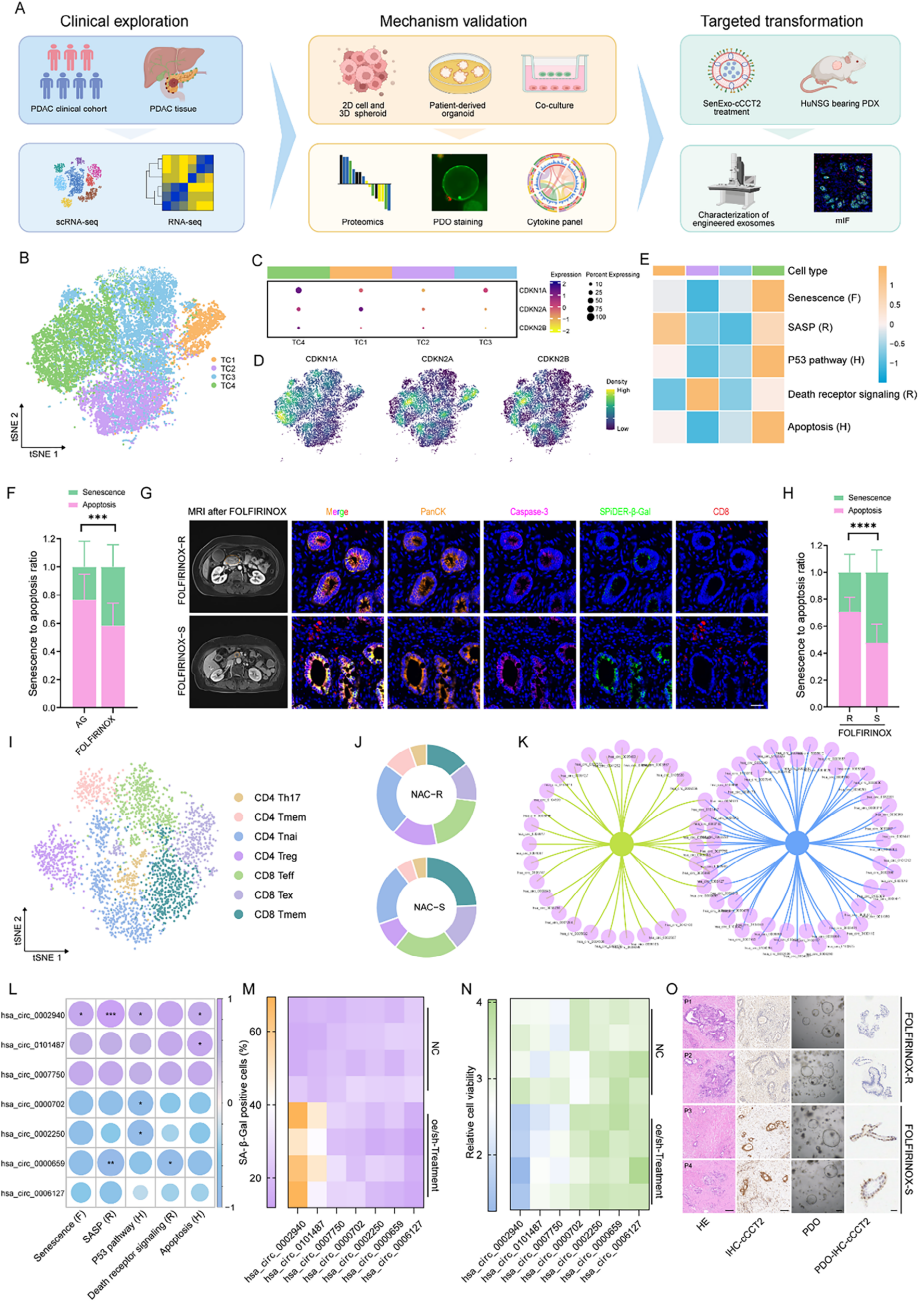
Correlation of tumor cell senescence with chemosensitivity and CD8^+^ T‐cell infiltration in PDAC A) The overall experimental scheme of this study. B) t‐SNE plots of the four tumor cell (TC) clusters sorted from the human PDAC scRNA‐seq data. C) Dot plot of senescence marker genes of the four tumor cell (TC) clusters, with the dot size representing the percent expression in the cluster and the dot color scaled by the expression level. D) t‐SNE plots showing the expression of senescence marker genes. E) GSVA showing the average GSVA score per cluster for senescence‐related pathways. Database: Fridman (F), Hallmark (H), Reactome (R). F) Comparison of the senescence‐apoptosis ratio (SAR) across chemotherapy regimens. G) Representative MRI and mIF images for FOLFIRINOX resistant (R) and sensitive (S) patients, demonstrating PDAC size, senescence and apoptosis distribution, and CD8^+^ T‐cell infiltration after chemotherapy. Orange circles: primary pancreatic tumors. Scale bar, 50 µm. H) Comparison of the SAR by response status within the FOLFIRINOX subgroup. I) t‐SNE plots of the seven identified T lymphocyte clusters sorted from the human PDAC scRNA‐seq data. J) The proportions of different defined T lymphocyte types in the T lymphocyte population. K) Network Venn diagram illustrating the overlap between differentially expressed circRNAs between the FOLFIRINOX‐R and FOLFIRINOX‐S groups and the senescence high‐ and low‐ groups. L) Dot plot demonstrating the correlation between the seven circRNAs screened and senescence‐related pathways, with dot size and dot color representing the correlation coefficient. M) Heat map depicting the percentage of SA‑β‑Gal‑positive cells following overexpression or knockdown of the seven candidate circRNAs. N) Heat map depicting relative cell viability following overexpression or knockdown of the seven candidate circRNAs. O) Representative images of H&E staining and cCCT2 immunohistochemistry in the FOLFIRINOX‐R and FOLFIRINOX‐S PDAC tissues and patient‐derived organoid (PDO). Data are expressed as the means ± SDs. Unpaired two‐tailed Student's t‐test or Mann–Whitney *U*‐test (F, H), Spearman analysis (L). Not significant (ns); ^*^ *P* < 0.05; ^**^ *P* < 0.01; ^***^ *P* < 0.001; ^****^ *P* < 0.0001. Figure 1A created with BioRender.com.

To characterize tumor cell dynamics and microenvironmental remodeling in PDAC following neoadjuvant chemotherapy, we performed scRNA‐seq using freshly dissociated tumor samples from patients with PDAC after AG or FOLFIRINOX treatment (**Figure** **1**A; Table S1, Supporting Information). These samples spanned the tumor, epithelial, stromal, and immune compartments (Figure S1A–F, Supporting Information). Subclustering revealed refined subpopulations of tumor cells (Figure 1B). Based on previous studies,^[^ ^33^, ^34^, ^35^ ^]^ we evaluated senescence marker genes, such as CDKN1A (p21), CDKN2A (p16), and CDKN2B (p15). A cluster with high expression of all three genes was identified and defined as senescent tumor cells (senTCs) (Figure 1C,D; Figure S1G,H, Supporting Information). Genomes associated with senescence‐associated pathways were used to enrich senTCs (Figure 1E). Notably, the proportion of senTCs was significantly greater in neoadjuvant chemotherapy‐sensitive (NAC‐S) patients, and this effect was more pronounced in patients treated with FOLFIRINOX (Figure S1I,J, Supporting Information). To assess the association between senTCs and different neoadjuvant chemotherapy regimens, we analyzed 96 patients (43 FOLFIRINOX vs 53 AG) and stratified the cohort into senescence‐high and senescence‐low groups based on the proportion of senescent‐positive cells. The high‐senescence group showed improved overall survival and recurrence‐free rates (Figure S1K,L, Supporting Information). Compared with AG, FOLFIRINOX induced a higher proportion of senTCs (Figure S1M, Supporting Information). Further subgroup analysis within the treatment regimens revealed that the prevalence of senescence was higher in the chemotherapy‐response subgroups across both chemotherapy regimens (Figure S1N, Supporting Information).

Given that apoptosis serves as a classical biomarker of the chemotherapy‐response (Figure S1O,P, Supporting Information), we hypothesized that cellular senescence could represent a potential indicator of chemosensitivity. We designed the senescence‐to‐apoptosis ratio (SAR) to assess the proportion of senescence within the combined senescence‐apoptosis indices. Notably, senescence was predominant in FOLFIRINOX‐treated group (Figure 1F). Subsequent subgroup analyses revealed significantly elevated SAR in chemotherapy‐sensitive populations compared to their resistant counterparts across both the FOLFIRINOX and AG regimens (Figure 1G,H; Figure S1Q, Supporting Information), supporting senescence as a predictive biomarker of chemosensitivity. Next, we observed changes in the tumor immune microenvironment after an increase in senTCs induced by chemotherapy, especially in terms of T‐cells, which are crucial for immune surveillance. Subclassification of T‐cell subpopulations revealed a more pronounced increase in CD8^+^ T‐cells in the chemotherapy‐responsive subgroups (Figure 1I,J). Multiplex immunofluorescence (mIF) analysis further revealed enhanced CD8^+^ T‐cell infiltration adjacent to senTCs (Figure 1G). Correlation analyses specifically linked CD8^+^ T‐cell infiltration to senescence (Figure S1R,S, Supporting Information).

To investigate the role of circRNAs in these chemotherapy‐responsive patients with a higher senTC burden, we selected the top three and bottom three patients ranked in order of senescent‐positive cells, along with three paired FOLFIRINOX resistant (R) and sensitive (S) patients for circRNA‐Seq analysis (Figure S2A–C, Supporting Information). Seven differentially expressed circRNAs were common between the two groups (Figure 1K). After correlation analysis of senescence‐related pathways and expression profiling of these circRNAs in PDAC cell lines (Figure 1L; Figure S2D, Supporting Information), we overexpressed the circRNAs positively correlated with senescence and apoptosis in cells with low expression and that knocked down circRNAs were negatively correlated with senescence and apoptosis in cells with high expression (Figure S2E, Supporting Information). Following SA‐β‐Gal and chemotherapy sensitivity assessment, we ultimately focused on cCCT2 (hsa_circ_0002940), which had the most significant effect on both senescence induction and chemosensitivity (Figure 1M,N). RT‐qPCR and ISH analyses revealed that cCCT2 expression was upregulated in chemotherapy‐responsive PDAC tissues and patient‐derived organoids (PDOs) (Figure 1O; Figure S2F, Supporting Information). In conclusion, an increased number of senTCs with increased CD8^+^ T‐cell infiltration is an important feature of chemosensitivity in patients with PDAC. cCCT2 may be a key molecule contributing to post‐chemotherapy senescence of PDAC cells and chemosensitization in PDAC patients.

### 2.2 cCCT2 Overexpression Mediates Cellular Senescence following FOLFIRINOX in PDAC

**Figure 2:**
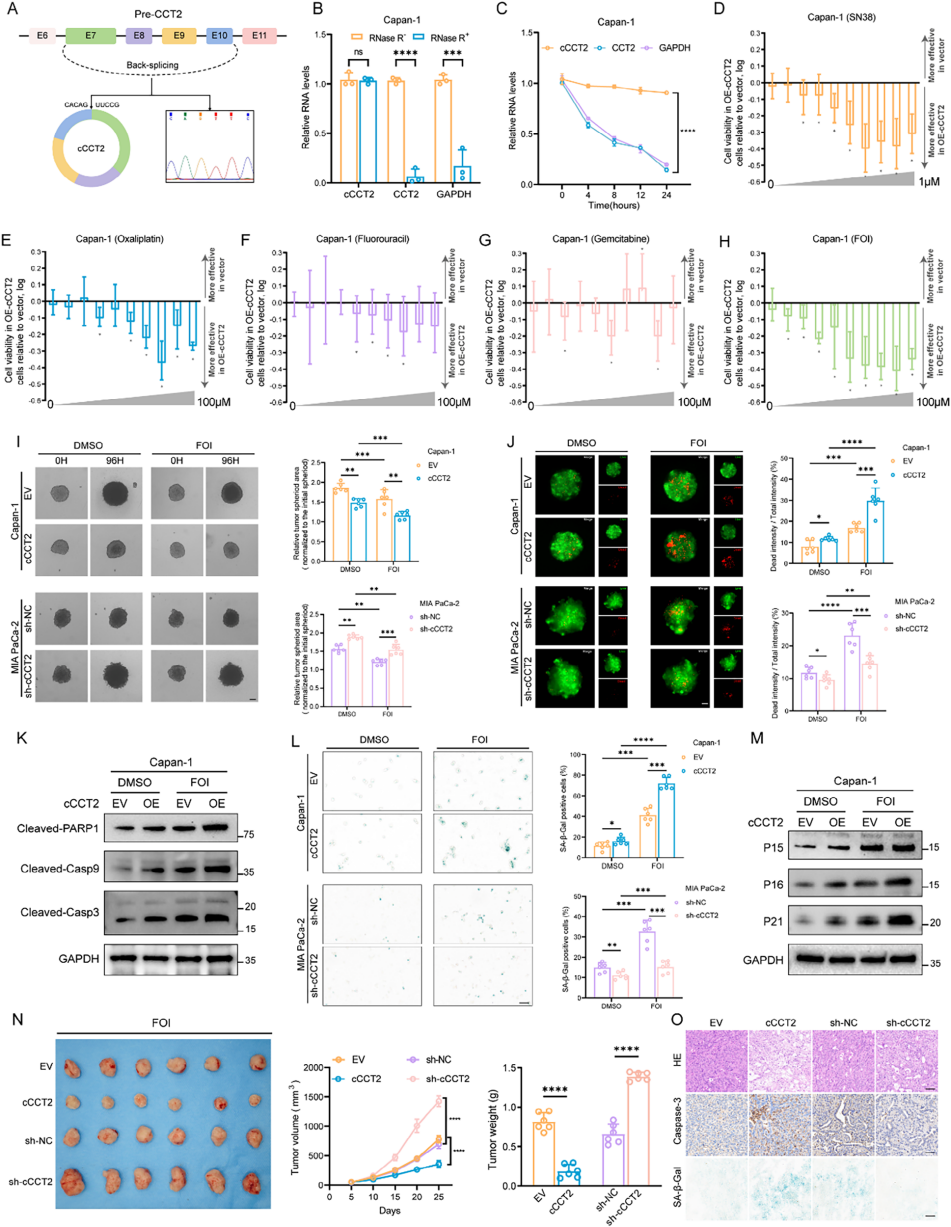
cCCT2 overexpression mediates cellular senescence following FOLFIRINOX in PDAC A) Schematic structure of cCCT2 and Sanger sequencing analysis of the back splicing junction in endogenously amplified cCCT2. B) RNA stability analysis of cCCT2, CCT2, and GAPDH in RNase R‐treated Capan‐1 cells. GAPDH mRNA was used as a negative control (n = 3 per group). C) RT–qPCR analysis of cCCT2 and CCT2 stability in Capan‐1 cells following actinomycin D treatment (n = 3 per group). GAPDH mRNA serves as negative control. D–H) Bar chart showing cell viability of EV‑ and OE‑cCCT2‑expressing Capan‐1 cells after treatment with the active metabolite of irinotecan SN38 (D), oxaliplatin (E), Fluorouracil (F), Gemcitabine (G), and three‐drug combination FOI (H) at the indicated concentrations (n = 6 per group). Bars show the ​log‐transformed viability ratio​ of cCCT2‐overexpressing cells relative to vector controls. Data display using mean with range. Bars extending below the baseline (0) indicate a reduction in viability of OE‑cCCT2 cells relative to EV controls. ^*^ *P* < 0.05. I) Representative images of the proliferation of Capan‐1 and MIA PaCa‐2 3D microtumor spheroids with cCCT2 overexpression or knockdown and quantification of the results (n = 6 per group). Scale bar, 100 µm. J) Representative images of the apoptosis of Capan‐1 and MIA PaCa‐2 3D microtumor spheroids following cCCT2 overexpression or knockdown and quantification of the results (n = 6 per group). Live cells were stained with calcein‐AM (green), and dead cells were stained with propidium iodide (PI) (red). Scale bar, 100 µm. K) Western blot analysis of apoptosis‑related protein expression in Capan‑1 cells overexpressing cCCT2 (n = 3). L) Representative images of SA‐β‐Gal‐staining in PDAC cells with different cCCT2 expression levels and quantification of the results (n = 6 per group). Scale bar, 30 µm. M) Western blot analysis of senescence‑related protein expression in Capan‑1 cells overexpressing cCCT2 (n = 3). N) Tumor volume growth curves and weights for xenograft nude mouse models generated with cCCT2‐overexpressing Capan‐1 and cCCT2‐knockdown MIA PaCa‐2 cells (n = 6 per group). O) Representative images of H&E staining, IHC staining for caspase‐3, and SA‐β‐Gal staining of tumor tissues from mice with cCCT2 overexpression or knockdown. Scale bar, 100 µm. Data are expressed as the means ± SDs. Unpaired two‐tailed Student's t‐test or Mann–Whitney *U*‐test (B), paired t‐test or Wilcoxon signed‐rank test (D–H), repeated measures ANOVA test (C, N), two‐way ANOVA with Tukey's post‐hoc test (I, J, L). Not significant (ns); ^*^ *P* < 0.05; ^**^ *P* < 0.01; ^***^ *P* < 0.001; ^****^ *P* < 0.0001.

The cCCT2 transcript is derived from the CCT2 transcript located on human chromosome 12 and is joined by back splicing of exons 7, 8, 9, and 10. Sanger sequencing confirmed that the head‐to‐tail splice junction sequences were consistent with annotated sequences (**Figure** **2**A). In addition, we designed divergent and convergent primers, and cCCT2 was amplified from cDNA using these divergent primers (Figure S3A, Supporting Information). RNase R and actinomycin D assays further confirmed the circular stability of cCCT2 (Figure 2B,C; Figure S3B,C, Supporting Information). Nuclear‐cytoplasmic fractionation combined with fluorescence in situ hybridization (FISH) revealed detectable cCCT2 levels in both the cytoplasm and nucleus, with slight nuclear enrichment (Figure S3D,E, Supporting Information). In addition, together with the clinical information of the patients, we found that high cCCT2 expression was associated with better prognosis (Figure S3F–I and Table S2, Supporting Information).

The cCCT2 transcript showed the highest expression in the MIA PaCa‐2 cell line and the lowest in Capan‐1 cells, which were subsequently selected as the experimental models (Figure S2E, Supporting Information). Initial treatment with individual FOLFIRINOX components (F, fluorouracil; O, oxaliplatin; I, the active metabolite of irinotecan SN38, and their combination FOI) revealed differential half‐maximal inhibitory concentrations (IC_50_) in Capan‐1 and MIA PaCa‐2 cell lines after 48 h drug exposure (Figure S4A, Supporting Information). Given the superior efficacy of the FOLFIRINOX combination therapy versus monotherapy, the concentration ranges were centered around the IC_25_ values of the constituent agents. To assess FOI sensitivity under differential cCCT2 expression, we generated stable cell lines with cCCT2 overexpression or short hairpin RNA (shRNA)‐mediated knockdown, confirming no compensatory alterations in linear CCT2 mRNA/protein levels, and selected sh#1 as the definitive knockdown tool (Figure S4B,C, Supporting Information). We found that cCCT2 modulation significantly sensitized cells to irinotecan, oxaliplatin, and fluorouracil, but minimally influenced gemcitabine susceptibility (Figure 2D–G). FOI combination treatment of cCCT2‐overexpressing Capan‐1 cells demonstrated dose‐dependent growth inhibitory effects (Figure 2H), suggesting that cCCT2 functionality is preferentially linked to FOI‐induced multifaceted DNA damage responses. Correspondingly, cell viability assays showed a 40.69% reduction in cCCT2‐overexpressing cells post‐48 h FOI treatment (Figure S4D, Supporting Information). EdU and 3D tumor spheroid proliferation assays confirmed that cCCT2 overexpression inhibited tumor cell proliferation under the influence of FOI, whereas the opposite effect was observed following cCCT2 knockdown (Figure 2I; Figure S4E, Supporting Information). The ability of cCCT2 to synergize with FOI was investigated by analyzing 3D tumor spheroid apoptosis and western blotting (Figure 2J,K; Figure S4F, Supporting Information). A significant number of cells with high cCCT2 expression were positively stained for SA‐β‐Gal (Figure 2L). These cells also showed elevated levels of P15, P16, and P21 (Figure 2M; Figure S4G, Supporting Information).

Finally, to explore the antitumor effect of cCCT2 in vivo and its effect on chemotherapy sensitivity, cCCT2‐overexpressing Capan‐1 and cCCT2‐knockdown MIA PaCa‐2 cells were injected into 6‐week‐old nude mice to establish a xenograft tumor model. Tumor volume and weight were lower in the cCCT2‐overexpressing group than in the control group. In contrast, the cCCT2 knockdown group exhibited a marked increase (Figure 2N). Immunohistochemistry (IHC) analysis revealed that the expression of caspase‐3 and SA‐β‐Gal‐positive cells was greater in the cCCT2‐overexpressing group (Figure 2O). Collectively, these results suggested that cCCT2 expression promotes senescence in PDAC cells and enhances their sensitivity to chemotherapy.

### 2.3 cCCT2 Overexpression Enhances FOLFIRINOX‐Induced Cellular Senescence via DNA Damage Accumulation

**Figure 3:**
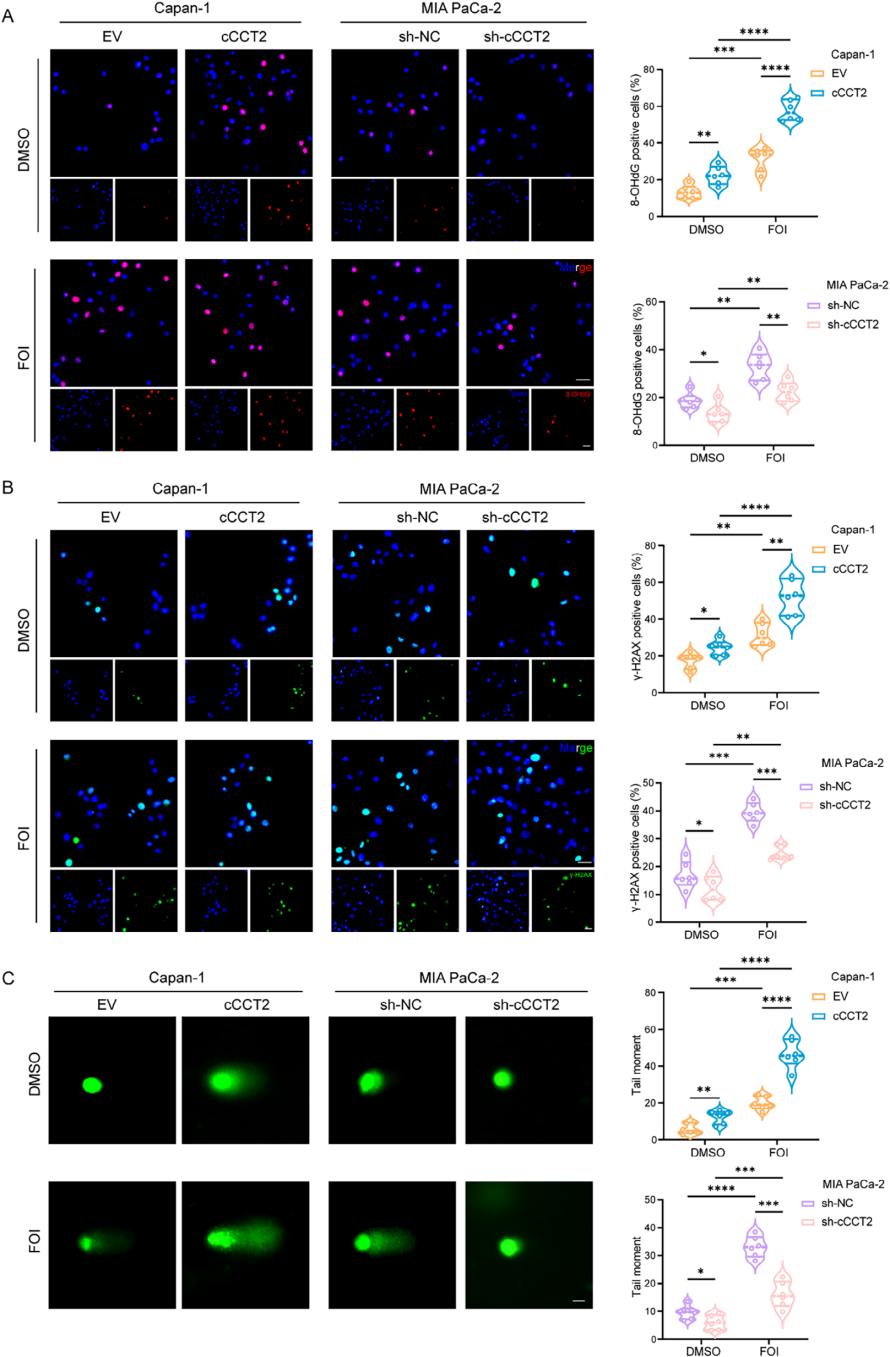
cCCT2 overexpression enhances FOLFIRINOX‐induced cellular senescence via DNA damage accumulation A) Representative images of 8‐OHdG accumulation in PDAC cells with different cCCT2 expression levels and quantification of the results (n = 6 per group). Scale bar, 30 µm. B) Representative images of γ‐H2AX accumulation in PDAC cells with different cCCT2 expression levels and quantification of the results (n = 6 per group). Scale bar, 30 µm. C) Comet assay detects DNA strand breaks in PDAC cells with different cCCT2 expression levels and quantification of the results (n = 6 per group). Scale bar, 10 µm. Two‐way ANOVA with Tukey's post‐hoc test (A–C). Not significant (ns); ^*^ *P* < 0.05; ^**^ *P* < 0.01; ^***^ *P* < 0.001; ^****^ *P* < 0.0001.

DNA damage serves as a shared trigger of cellular senescence and apoptosis. The accumulation of irreparable DNA damage may induce senescence, whereas excessive DNA damage activates apoptotic pathways. Therefore, we examined the effect of CCCT2 on DNA damage. In cCCT2‐overexpressing cells, both DMSO‐treated controls and FOI‐treated groups for 48 h exposure, exhibited upregulation of DNA damage markers (8‐OHdG, γ‐H2A.X) (**Figure** **3**A,B). Comet assays further confirmed elevated DNA tail moments in cCCT2‐overexpressing cells compared to those in controls. Conversely, cCCT2 knockdown attenuated the FOI‐induced DNA damage in MIA PaCa‐2 cells (Figure 3C). These findings established that DNA damage accumulation is the central mechanism underlying cCCT2‐mediated senescence and apoptosis in PDAC cells following chemotherapy.

### 2.4 cCCT2 Overexpression Potentiates DNA Damage Accumulation via Suppression of SLX4‐Mediated DNA Damage Repair Response

**Figure 4:**
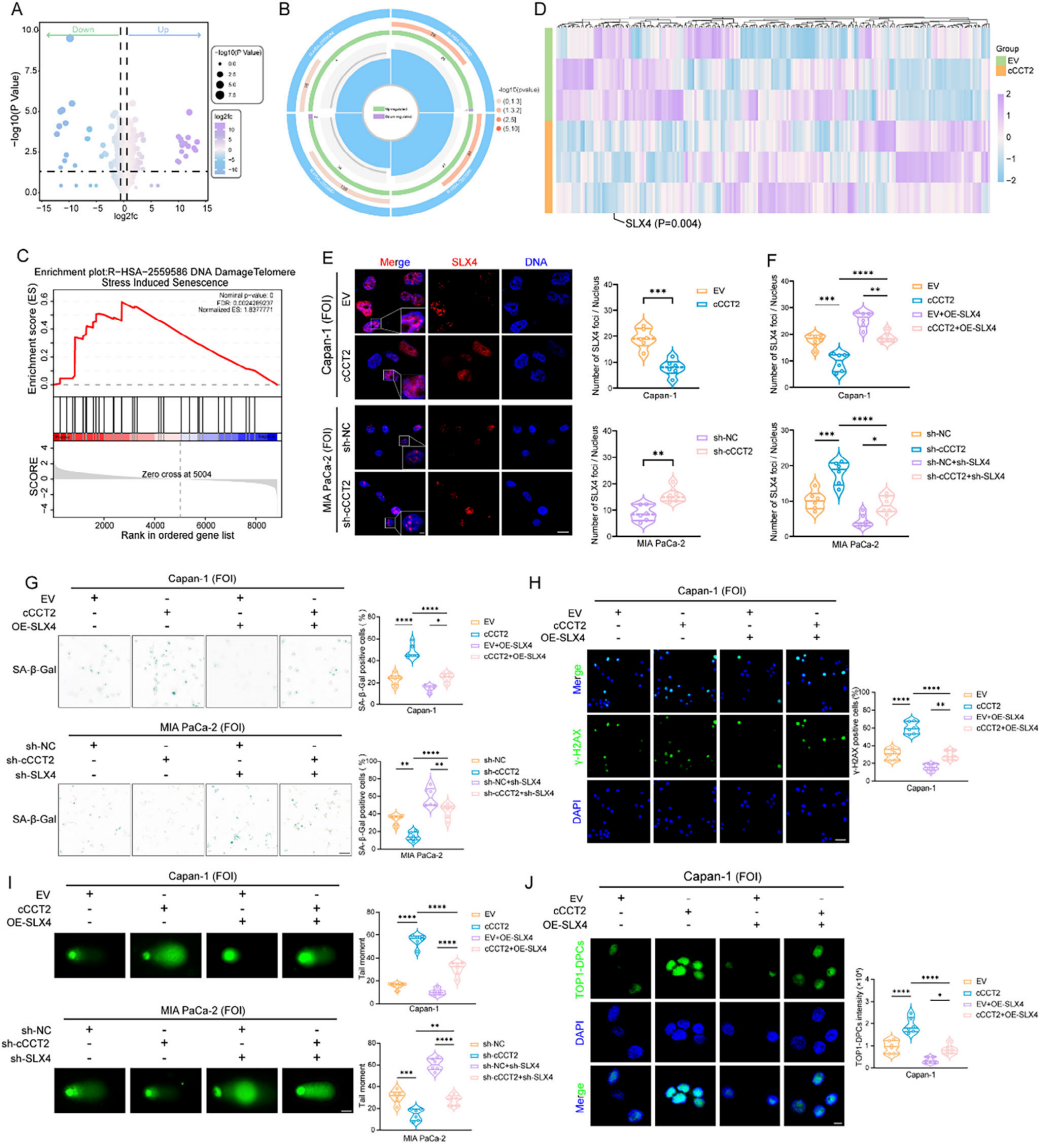
cCCT2 overexpression potentiates DNA damage accumulation via suppression of SLX4‐mediated DNA damage repair response A) Volcano plot of differentially expressed proteins in Capan‐1 with EV or overexpressing cCCT2 after FOI treatment. The dot color indicates protein expression after normalization, and the dot size represents the P value. B) Enrichment circle plot of the proteomic data showing the enrichment of senescence‐ and DDR‐related pathways. C) GSEA plot showing enrichment of the DNA damage/telomere stress‐induced senescence pathway in the Capan‐1 group with high cCCT2 expression levels. D) Heatmap of the differential expression of 276 DDR‐related proteins identified via proteomics. E) Representative images of SLX4 condensate formation in PDAC cells with different cCCT2 expression levels and quantification of the results (n = 6 per group). Scale bar, 20 µm. F) Quantification of the effect of altering SLX4 expression levels on SLX4 condensate formation in PDAC cells with different cCCT2 expression levels (n = 6 per group). G) SA‐β‐Gal staining showing the effect of altering SLX4 expression in PDAC cells with different cCCT2 expression levels and quantification of the results (n = 6 per group). Scale bar, 30 µm. H) Representative images of the effect of altering SLX4 expression levels on γ‐H2AX accumulation in Capan‐1 cells with EV or overexpressing cCCT2 and quantification of the results (n = 6 per group). Scale bar, 30 µm. I) Representative images of the effect of altering SLX4 expression levels on tail moment of comet assay in PDAC cells with different cCCT2 expression levels and quantification of the results (n = 6 per group). Scale bar, 20 µm. J) Representative images of the effect of altering SLX4 expression levels on TOP1‐DPCs staining in PDAC cells with different cCCT2 expression levels and quantification of the results (n = 6 per group). Scale bar, 20 µm. Unpaired two‐tailed Student's t‐test or Mann–Whitney *U*‐test (E). one‐way ANOVA with Tukey's post‐hoc test (F–J). Not significant (ns); ^*^ *P* < 0.05; ^**^ *P* < 0.01; ^***^ *P* < 0.001; ^****^ *P* < 0.0001.

To delineate the mechanistic basis of cCCT2‐driven senescence in PDAC cells, we performed proteomic profiling of FOI‐induced senTCs that overexpressed cCCT2 (**Figure** **4**A). Compared with the control group, senescence‐ and DDR‐related pathways were enriched in the cCCT2‐overexpressing group (Figure 4B). Therefore, we hypothesized that cCCT2 overexpression exacerbates DNA repair deficiencies, thereby accelerating senescence. Gene set enrichment analysis (GSEA) revealed that high levels of cCCT2 were more likely to lead to DNA damage or telomere stress‐induced senescence (Figure 4C). Differential expression screening of 276 DDR‐associated proteins highlighted SLX4, a multiple regulator of genomic stability, as the key mediator (Figure 4D). Western blot analysis validated reduced SLX4 expression in chemotherapy‐sensitive patients and cCCT2‐overexpressing cells (Figure S5A,B, Supporting Information).

SLX4 serves as a scaffold to drive the compartmentalization of DNA damage response proteins through phase separation.^[^ ^26^ ^]^ Confocal microscopy revealed that cCCT2 overexpression inhibited the formation of SLX4 nuclear foci (Figure 4E). To confirm that SLX4 is a critical downstream effector of cCCT2, we co‐overexpressed SLX4 and cCCT2 in Capan‐1 cells. Remarkably, SLX4 overexpression rescued the cCCT2‐mediated impairment of biomolecular condensate assembly, reversing cCCT2‐driven chemosensitization and senescence induction (Figure 4F,G; Figure S5C–E, Supporting Information). γ‐H2AX staining and comet assays further revealed that cCCT2 decreases the DNA repair capacity driven by SLX4 overexpression (Figure 4H,I; Figure S5F, Supporting Information). Additionally, cCCT2 attenuated SLX4 condensate‐mediated degradation of topoisomerase I‐DNA‒protein crosslinks (TOP1‐DPCs), as well as the repair efficiency of homologous recombination (HR), ICL, and non‐homologous end joining (NHEJ) (Figure 4J; Figure S5G–J, Supporting Information). Collectively, these findings established that SLX4 is a pivotal molecular target through which cCCT2 promotes DNA damage accumulation, senescence, and chemosensitivity in PDAC cells by dysregulating DDR‐associated SLX4 condensate dynamics.

### 2.5 cCCT2 Overexpression Suppresses SLX4‐Mediated DNA Damage Repair Response via Regulating SUMOylation

**Figure 5:**
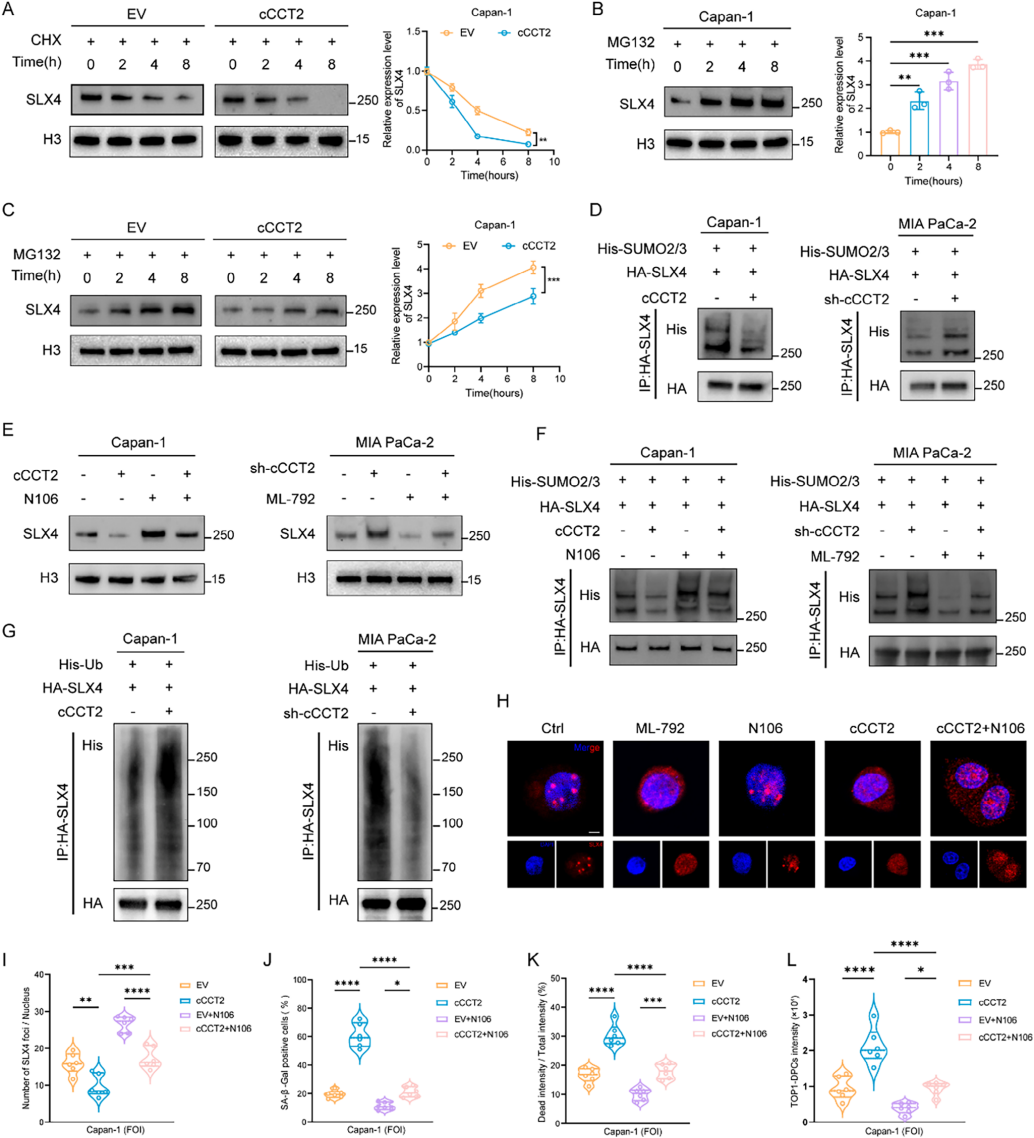
cCCT2 overexpression suppresses SLX4‐mediated DNA damage repair response via regulating SUMOylation A) Degradation of the SLX4 protein in Capan‑1 cells with EV or overexpressing cCCT2 after treatment with cycloheximide (CHX) for 2, 4, and 8 h and quantification of the half‐life (n = 3). B) Degradation of the SLX4 protein in Capan‐1 cells treated with MG132 for 2 h, 4 h, or 8 h and quantification of the results (n = 3). C) Degradation of the SLX4 protein in Capan‑1 cells with EV or overexpressing cCCT2 after treatment with MG132 for 2, 4, and 8 h and quantification of the results (n = 3). D) Western blot analysis of SLX4 SUMOylation levels at different cCCT2 expression levels. E) Western blot analysis of SLX4 levels at different cCCT2 expression levels following treatment with the SUMOylation agonist N106 and the SUMOylation inhibitor ML‐792 (n = 3). F) Western blot analysis of SLX4 SUMOylation levels at different cCCT2 expression levels following treatment with the SUMOylation agonist N106 and the SUMOylation inhibitor ML‐792 (n = 3). G) Western blot analysis of SLX4 ubiquitination levels at different cCCT2 expression levels (n = 3). H) Representative images illustrating the nuclear and cytoplasmic distribution of SLX4 in Capan‐1 cell with overexpressing cCCT2(n = 3). Scale bar, 10 µm. I) Assessment of the rescue effect of N106 on SLX4 condensate formation in Capan‑1 cells with EV or overexpressing cCCT2 (n = 6 per group). J) Assessment of the rescue effect of N106 on SA‐β‐Gal staining in Capan‑1 cells with EV or overexpressing cCCT2 (n = 6 per group). K) Assessment of the rescue effect of N106 on apoptosis of Capan‐1 3D microtumor spheroids with EV or overexpressing cCCT2 (n = 6 per group). L) Assessment of the rescue effect of N106 on TOP1‑DPCs accumulation in Capan‑1 cells with EV or overexpressing cCCT2 (n = 6 per group). Repeated measures ANOVA test (A, C), one‐way ANOVA with ​Dunnett's post‐hoc test (B), one‐way ANOVA with Tukey's post‐hoc test (I–L). Not significant (ns); ^*^ *P* < 0.05; ^**^ *P* < 0.01; ^***^ *P* < 0.001; ^****^ *P* < 0.0001.

To elucidate how cCCT2 modulates SLX4 expression and biomolecular condensate dynamics, we performed AGO2 RNA immunoprecipitation (RIP) assays and FLAG‐tagged immunoprecipitation (IP) experiments, which ruled out potential competitive endogenous RNA (ceRNA) mechanisms or the peptide‐encoding potential of cCCT2 (Figure S6A–C, Supporting Information). In the scRNA‐seq data, SLX4 mRNA expression levels did not show significant alterations in senTCs (Figure S6D, Supporting Information). Critically, cCCT2 knockdown or overexpression regulated SLX4 at the protein level without altering its mRNA levels (Figures S5B and S6E, Supporting Information), indicating the post‐translational regulation of SLX4 by cCCT2 in PDAC cells. To investigate SLX4 degradation dynamics, we treated cells with cycloheximide (CHX) for 2, 4, or 8 h to block de novo protein synthesis. Western blotting analysis revealed that SLX4 protein levels decreased more rapidly over time in the high cCCT2 expression group than in the control group, whereas SLX4 degradation was slower in the cCCT2 knockdown group (**Figure** **5**A; Figure S6F, Supporting Information). To determine which pathway is involved in SLX4 degradation, we treated cells with the lysosomal degradation pathway inhibitor chloroquine (CQ) and the proteasomal degradation pathway inhibitor MG132 for 2, 4, and 8 h. Western blot analysis revealed that the amount of SLX4 protein in the MG132‐treated group gradually increased, while CQ treatment had no notable effect on total SLX4 levels. (Figure 5B; Figure S6G–I, Supporting Information), indicating that the degradation of SLX4 occurs mainly through the proteasome pathway. Next, we treated cells with different cCCT2 expression levels with MG132, and cCCT2 overexpression slowed SLX4 accumulation under MG132 treatment (Figure 5C; Figure S6J, Supporting Information).

Small ubiquitin‐like modifier (SUMO) modifications, known as SUMOylation, can stabilize protein levels through various pathways and regulate nucleocytoplasmic distribution by modulating nuclear import/export. Notably, numerous DDR‐associated proteins undergo SUMOylation during repair processes, and SLX4 has been identified as a SUMOylation substrate.^[^ ^36^, ^37^ ^]^ SUMOylation assays confirmed that cCCT2 overexpression decreased SLX4 SUMOylation, whereas cCCT2 knockdown increased it (Figure 5D). Pharmacological modulation using the SUMOylation agonist N106 restored SLX4 protein levels and SUMOylation was diminished by cCCT2, whereas the SUMOylation inhibitor ML‐792 counteracted the effects of cCCT2 knockdown (Figure 5E,F). Western blotting revealed that cCCT2 overexpression increased SLX4 ubiquitination, whereas cCCT2 knockdown reduced global SLX4 ubiquitination (Figure 5G). Fluorescence localization assays showed that the cytoplasmic distribution of SLX4 was increased by either ML‐792 or cCCT2 overexpression, and that this effect was reversed by N106 (Figure 5H), suggesting that cCCT2‐mediated SUMOylation loss drives SLX4 nuclear export and subsequent ubiquitin‐proteasomal degradation. Cellular function experiments demonstrated that modulation of SUMOylation reversed the effects of cCCT2 on biomolecular condensate formation, chemosensitivity, and senescence in PDAC cells (Figure 5I–L; Figure S6K–R, Supporting Information). In conclusion, cCCT2 regulates the proteasomal degradation pathway and condensate formation of SLX4 via SUMOylation. However, the precise mechanism underlying this interaction requires further investigation.

### 2.6 cCCT2 Overexpression Modulates SUMOylation of SLX4 via Competitive Binding to IPO13

**Figure 6:**
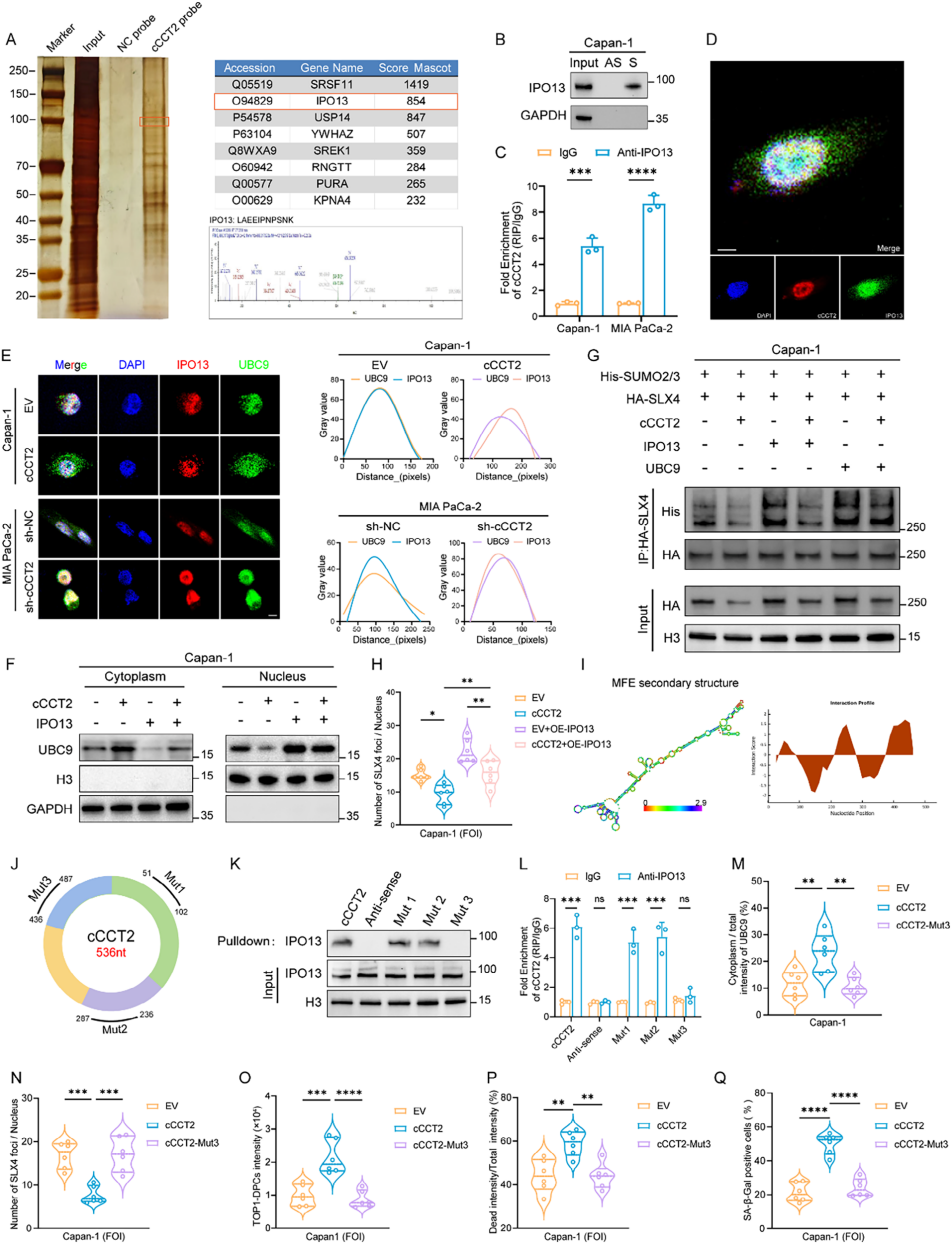
cCCT2 overexpression modulates SUMOylation of SLX4 via competitive binding to IPO13 A) Identification of RBP binding to specific biotin‐labeled cCCT2 (sense) and control (antisense) probes via silver staining and mass spectrometry. Labeled region of the strip corresponding to IPO13 (97 kDa). B) Interaction between cCCT2 and the IPO13 protein was detected in Capan‐1 cells via a specific biotin‐labeled cCCT2 (sense) RNA pull‐down assay. C) RT‐qPCR was performed after the RIP experiments to detect the amount of cCCT2 bound to the IPO13 protein (n = 3). D) Colocalization of cCCT2 (red) and IPO13 (green) was determined via RNA‐FISH and immunofluorescence staining, and the cell nuclei were stained with DAPI (blue). Scale bar, 10 µm. E) Colocalization of UBC9 (green) and IPO13 (red) in PDAC cells with different cCCT2 expression levels and quantification of the results. Scale bar, 30 µm. F) Assessment of nuclear and cytoplasmic distribution of UBC9 in Capan‑1 cells with EV or overexpressing cCCT2 following altering IPO13 expression levels. H3 was used as a nuclear marker, and GAPDH was used as a cytoplasmic marker. G) Measurement of SLX4 protein expression and SUMOylation levels in Capan‑1 cells with EV or overexpressing cCCT2 following the overexpression of IPO13 or UBC9. H) Assessment of the effect of altering IPO13 expression levels on SLX4 condensation formation in Capan‑1 cells with EV or overexpressing cCCT2 (n = 6 per group). I) Prediction of the structural regions on cCCT2 that can bind to IPO13 via the bioinformatics tools RNAfold and catRAPID. J) Design of mutations that disrupt the candidate binding site of cCCT2 to IPO13. K) RNA pull‐down assay to assess the binding between three cCCT2 mutants and IPO13 protein. L) RT‒qPCR after the RIP assay to assess the binding of three cCCT2 mutants and IPO13 protein (n = 3 per group). M) Assessment of the effect of cCCT2‐Mut 3 on the nuclear and cytoplasmic distribution of UBC9 in Capan‐1 cells (n = 6 per group). N) Assessment of the effect of cCCT2‐Mut 3 on SLX4 condensation formation in Capan‐1 cells (n = 6 per group). O) Assessment of the effect of cCCT2‐Mut 3 on TOP1‑DPCs accumulation in Capan‐1 cells (n = 6 per group). P) Assessment of the effect of cCCT2‐Mut 3 on apoptosis of Capan‐1 3D microtumor spheroids (n = 6 per group). Q) Assessment of the effect of cCCT2‐Mut 3 on SA‐β‐Gal staining in Capan‐1 cells (n = 6 per group). Unpaired two‐tailed Student's t‐test or Mann–Whitney *U*‐test (C, L), one‐way ANOVA with Dunnett's post‐hoc test (M–Q), one‐way ANOVA with Tukey's post‐hoc test (H). Not significant (ns); ^*^ *P* < 0.05; ^**^ *P* < 0.01; ^***^ *P* < 0.001; ^****^ *P* < 0.0001.

To clarify how cCCT2 regulates the SUMOylation of SLX4, we performed an RNA pull‐down assay, which revealed that cCCT2 did not directly bind to SLX4 (Figure S7A, Supporting Information). The cCCT2 pull‐down products were analyzed using mass spectrometry; however, no key molecules directly associated with SUMOylation were identified. Further RIP assays targeting classical SUMO‐related proteins did not detect any interaction between CCT2 and components of the SUMOylation pathway (Figure S7B, Supporting Information). However, we detected Importin‐13 (IPO13) (**Figure** **6**A), a bidirectional karyopherin that recognizes and mediates the entry of ubiquitin‐conjugating enzyme 9 (UBC9), a SUMO E2 conjugation enzyme, into the nucleus.^[^ ^38^ ^]^ Molecular docking between cCCT2 and IPO13 was simulated using the HDOCK server^[^ ^39^ ^]^ (Figure S7C, Supporting Information), and the interaction was further validated by RNA pull‐down, RIP, crosslinking immunoprecipitation (CLIP), and IF‐FISH assays (Figure 6B–D; Figure S7D, Supporting Information). Additionally, RT‐qPCR and western blotting revealed that cCCT2 did not affect IPO13 or UBC9 mRNA or protein expression (Figure S7E,F, Supporting Information). Moreover, an immunoprecipitation assay revealed that SLX4 did not bind to IPO13 (Figure S7G, Supporting Information). Therefore, we hypothesized that cCCT2 affects SUMOylation of the SLX4 condensate by competitively binding to IPO13, which in turn reduces UBC9 entry into the nucleus.

To assess whether cCCT2 regulates the nuclear import of UBC9, a nuclear‐cytoplasmic fractionation assay was performed, which revealed that cCCT2 impedes the entry of UBC9 into the nucleus and that knockdown of cCCT2 increased its entry (Figure S7H, Supporting Information). Immunoprecipitation and immunofluorescence experiments revealed that UBC9 binding to IPO13 was significantly reduced after cCCT2 overexpression (Figure 6E; Figure S7I,J, Supporting Information). Strikingly, IPO13 knockdown or overexpression did not affect total UBC9 levels but affected cCCT2‐driven alterations in nuclear UBC9 (Figure 6F; Figure S7K,L, Supporting Information). Conversely, overexpression of IPO13 and UBC9 rescued cCCT2‐mediated SLX4 SUMOylation and restored condensate formation (Figure 6G,H; Figure S7M–P, Supporting Information). These findings further confirm that the key link in the regulation of SUMOylation by cCCT2 lies in the blockade of UBC9 entry into the nucleus.

To explore how cCCT2 affects the function of IPO13, we used the bioinformatics tools RNAfold and catRAPID, which predicted that the three well‐characterized stem‐loop junctions in cCCT2 could bind to IPO13 (Figure 6I). We mutated each of the potentially binding segments: Mut 1 (51‐102 nt), Mut 2 (236‐287 nt), and Mut 3 (436‐487 nt) (Figure 6J; Figure S8A, Supporting Information), while ensuring that the circular structure was maintained and minimizing alterations to the secondary stem‐loop structure as much as possible (Figure S8B,C, Supporting Information). RNA pull‐down and RIP analyses revealed that only Mut3 attenuated cCCT2 binding to IPO13 without compromising UBC9 nuclear import (Figure 6K–M; Figure S8D, Supporting Information). Cellular functional experiments investigated the critical role of nucleotides 436–487 in the formation of SLX4 condensates, the DDR process, cellular senescence, and chemosensitivity (Figure 6N–Q; Figure S8E, Supporting Information). Consistent findings were obtained in vivo using a mouse xenograft tumor model (Figure S8F,G, Supporting Information). Overall, these data confirm that binding to IPO13 via nucleotides 436–487 is essential for the ability of cCCT2 to promote cellular senescence and chemosensitivity in PDAC.

### 2.7 cCCT2 Overexpression Drives CD8^+^ T‐Cell Infiltration but Upregulates PD‐L1 in Tumor Cells

**Figure 7:**
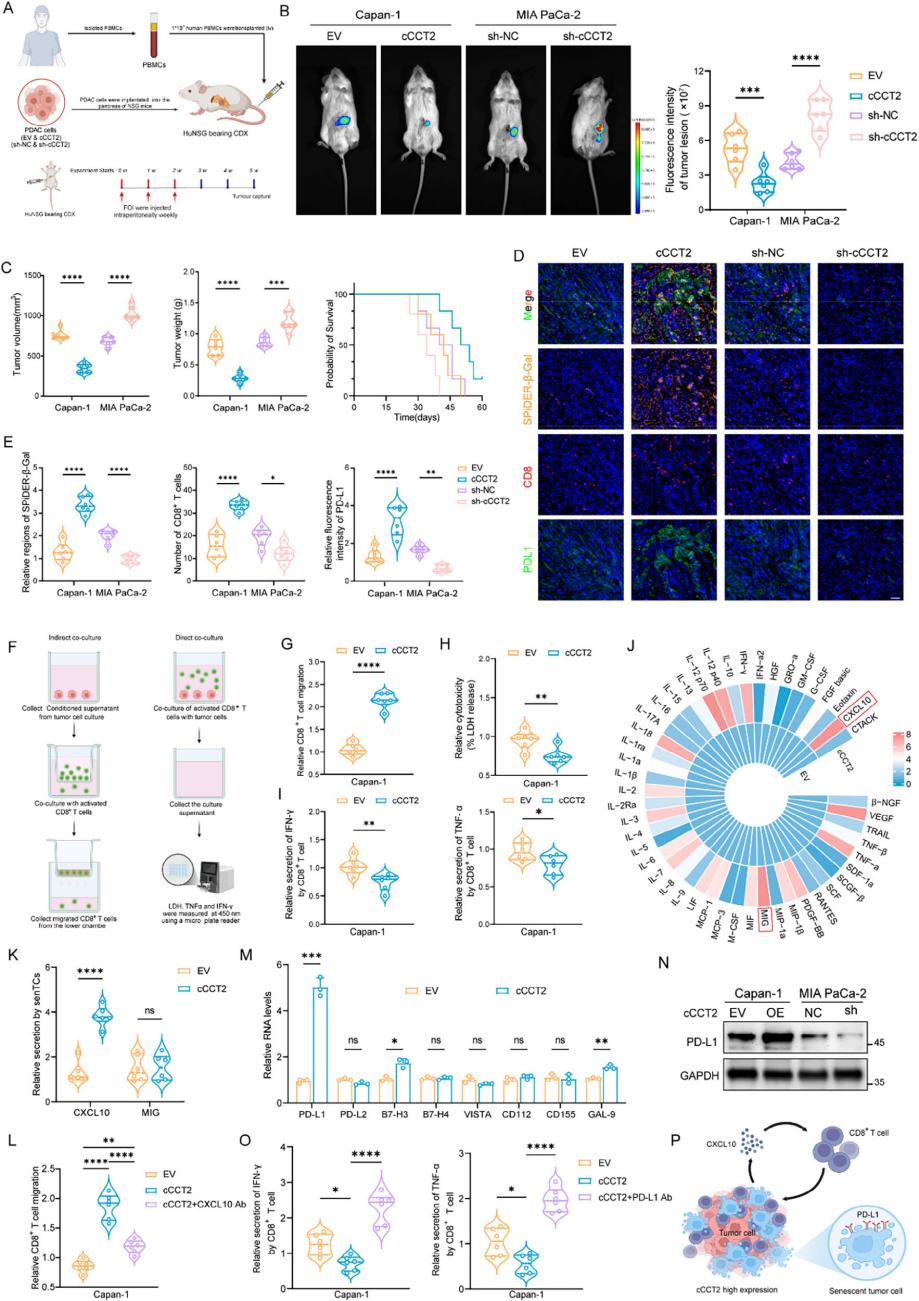
cCCT2 overexpression drives CD8^+^ T‐cell infiltration but upregulates PD‐L1 in tumor cells A) Diagrammatic drawing of the establishment of pancreatic xenograft tumor mouse model with a humanized immune system. B) Representative bioluminescence images and quantification of the bioluminescence intensity of tumors with different cCCT2 expression levels from Hu‐NSG mice (n = 6 per group). C) Tumor volume, tumor weights, and survival curves for xenograft mouse models generated with cCCT2‐overexpressing Capan‐1 and cCCT2‐knockdown MIA PaCa‐2 cells (n = 6 per group). D) Representative mIF images of in situ pancreatic tumors with different cCCT2 expression levels from Hu‐NSG mice. Scale bar, 100 µm. E) Quantification of SPiDER‐β‐Gal, CD8, and PD‐L1 expression in tumor mIF images (n = 6 per group). F) Schematic diagram of the co‐culture system between activated CD8⁺ T‐cells and Capan‑1 cells with EV or overexpressing cCCT2 after FOI treatment. G) Chemotaxis of CD8^+^ T‐cells induced by Capan‐1 cells with EV or overexpressing cCCT2 (n = 6 per group). H) Cytotoxicity mediated by CD8^+^ T‐cells against Capan‐1 cells with EV or overexpressing cCCT2, as measured by lactate dehydrogenase (LDH) release (n = 6 per group). I) ELISAs of IFN‐γ and TNF‐α levels in the culture supernatants of CD8⁺ T‐cells co‑cultured with Capan‑1 cells with EV or overexpressing cCCT2 (n = 6 per group). J) Cyclic heatmap of cytokines in culture supernatants of Capan‐1 cells with EV or overexpressing cCCT2 detected via the 48‐Plex Bio‐Plex Pro Human Cytokine Screening Panel. K) ELISAs of CXCL10 and MIG levels in Capan‐1 cells with EV or overexpressing cCCT2 after FOI treatment (n = 6 per group). L) Chemotaxis of Capan‐1 cells with EV or overexpressing cCCT2 toward CD8^+^ T‐cells in response to anti‐CXCL10 neutralizing antibody blockade (n = 6 per group). M) Expression levels of common immune checkpoint in Capan‐1 cells with EV or overexpressing cCCT2 after FOI treatment (n = 3 per group). N) Western blot analysis of PD‐L1 expression in Capan‑1 cells with EV or overexpressing cCCT2 after FOI treatment. O) ELISAs of IFN‐γ and TNF‐α levels in the culture supernatants of CD8⁺ T‐cells co‑cultured with Capan‑1 cells with EV or overexpressing cCCT2 under anti‐PD‐L1 antibody blockade (n = 6 per group). P) Schematic illustration of the immunomodulatory mechanisms between senescent tumor cells and CD8^+^ T‐cells. Unpaired two‐tailed Student's t‐test or Mann–Whitney *U*‐test (B, C, E, G–I, K, M). one‐way ANOVA with Tukey's post‐hoc test (L, O). Log rank test was used to compare the survival time. Not significant (ns); ^*^ *P* < 0.05; ^**^ *P* < 0.01; ^***^ *P* < 0.001; ^****^ *P* < 0.0001. A, F, P created with BioRender.com.

Senescent cells may influence the infiltration of CD8^+^ T‐cells via SASP. Therefore, we further investigated how cCCT2 alters CD8^+^ T‐cell infiltration following the induction of tumor cell senescence. We established a humanized immune system in immunodeficient NSG mice by introducing human peripheral blood mononuclear cells (PBMCs) (**Figure** **7**A). Following cCCT2 overexpression, the growth of PDAC xenografts was significantly inhibited (Figure 7B,C). Notably, this suppression correlated with increased senTC accumulation and markedly increased intratumoral infiltration of CD8^+^ T‐cells (Figure 7D,E, left and middle). To delineate the interplay between senTCs and CD8^+^ T‐cells, we co‐cultured chemotherapy‐induced senescent cCCT2‐overexpressing Capan‐1 cells with peripheral blood CD8^+^ T‐cells isolated from human donors (Figure 7F). cCCT2‐overexpressing cells induced CD8^+^ T‐cell migration to a greater extent than control cells (Figure 7G). Paradoxically, cytotoxicity assays revealed attenuated CD8^+^ T‐cell‐mediated tumor cell killing against cCCT2‐overexpressing targets, concomitant with reduced IFN‐γ and TNF‐α levels in co‐culture supernatants (Figure 7H,I).

Next, we explored the factors leading to the increased infiltration of CD8^+^ T‐cells in the microenvironment of patients with pancreatic cancer with high cCCT2 expression. Using a human cytokine screen, we identified CXCL10 and MIG (CXCL9) as the secreted cytokines whose levels were significantly increased in cCCT2 overexpression PDAC cells after FOI treatment (Figure 7J). Single‐cell data analysis and ELISA further confirmed the upregulation of CXCL10 (Figure 7K; Figure S9A, Supporting Information). Notably, CXCL10 is a chemoattractant of CD8^+^ T‐cells.^[^ ^40^ ^]^ Using CellChat analysis, we further confirmed significant intercellular communication between senTCs and CD8⁺ T‐cells, with the CXCL10‐CXCR3 ligand‐receptor axis identified as a key signaling pathway involved in this interaction (Figure S9B,C, Supporting Information). Rescue experiments using neutralizing antibodies in coculture systems demonstrated that anti‐CXCL10 antibodies largely abrogated senTC‐mediated chemotactic effects (Figure 7L) yet failed to restore cytotoxic function (Figure S9D–F, Supporting Information). However, the scRNA‐seq and experimental results described above revealed that, despite the increased infiltration of CD8^+^ T‐cells, the exhaustion phenotype was enhanced in vivo (Figures 1J and 7H,I). We examined the common immune checkpoints and found that PD‐L1 expression was significantly upregulated in FOI‐induced senTCs overexpressing cCCT2 (Figure 7M). Immunoblotting and immunofluorescence confirmed these results (Figure 7D,N, and Figure 7E right). Anti‐PD‐L1 antibody intervention restored CD8^+^ T‐cell‐mediated tumor cytotoxicity (Figure 7O; Figure S9G, Supporting Information). In summary, the senescence‐inducing effect of cCCT2 exhibits a dual‐edged nature. While the SASP facilitates CD8^+^ T‐cell recruitment, concomitant PD‐L1 upregulation in senTCs enables immune escape (Figure 7P). Therefore, we hypothesized that inducing senescence in tumor cells, combined with senolytic immunotherapy, represents a potential strategy for enhancing the efficacy of chemotherapy in pancreatic cancer.

### 2.8 SenExo‐cCCT2 Synergizes with Chemotherapy and Sequential Anti‐PD‐L1 Therapy to Suppress Pancreatic Cancer Progression

**Figure 8:**
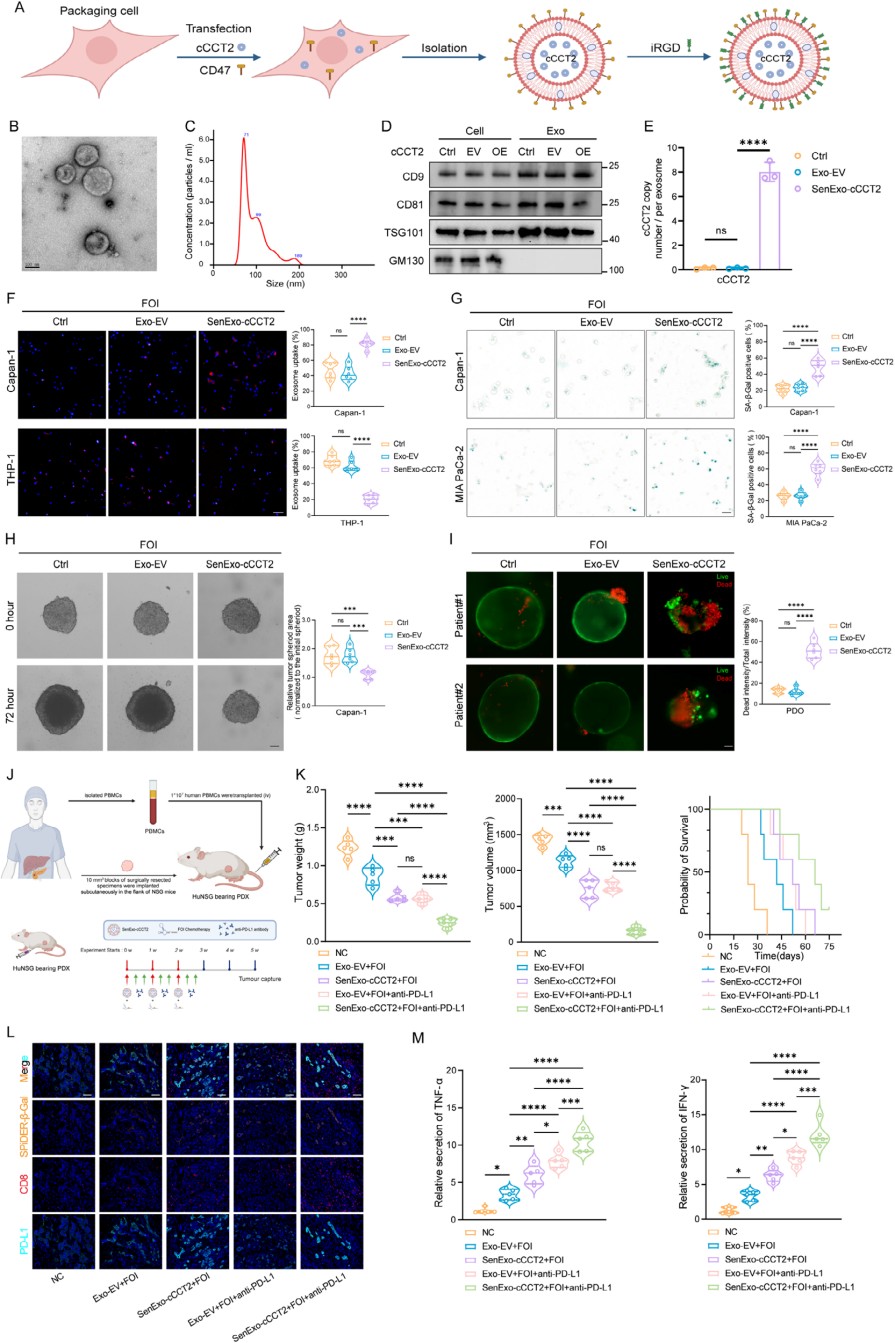
SenExo‐cCCT2 synergizes with chemotherapy and sequential anti‐PD‐L1 therapy to suppress pancreatic cancer progression A) Schematic diagram of the exosome‐based delivery system used to construct SenExo‐cCCT2. B) Representative transmission electron microscopy images of SenExo‐cCCT2. Scale bar, 100 nm. C) Size distribution of the SenExo‐cCCT2 analyzed with a nanolaser particle detector. D) Western blot analysis of inclusion and exclusion exosome markers. E) Average cCCT2 copies per exosome as determined by absolute qPCR (n = 6 per group). F) Representative fluorescence microscopy images of DiI‐labeled (red) exosomes taken up by PDAC cells (Capan‐1) and macrophages (THP‐1) (n = 6 per group). The cell nuclei were stained with Hoechst (blue). Scale bar, 50 µm. G) SA‐β‐Gal staining of PDAC cells after SenExo‐cCCT2 intervention and quantification of the results (n = 6 per group). Scale bar, 30 µm. H) Assessment of the effect of SenExo‐cCCT2 intervention on the proliferation of Capan‐1 3D microtumor spheroids derived from cells (n = 6 per group). Scale bar, 100 µm. I) Assessment of the effect of SenExo‐cCCT2 intervention on the apoptosis of patient‐derived organoids (PDO) and quantification of the results (n = 6 per group). Live cells were stained with calcein‐AM (green), and dead cells were stained with PI (red). Scale bar, 50 µm. J) Schematic diagram of the patient‐derived xenograft (PDX) model established on the basis of a humanized immune system model in NSG mice. K) Tumor volume, tumor weight, and survival curves of Hu‐NSG bearing PDX models treated with different combinations of SenExo‐cCCT2, chemotherapy, and anti‐PD‐L1 antibodies (n = 5 per group). L) Representative mIF images of tumors from Hu‐NSG bearing PDX models after treatment with different combinations of SenExo‐cCCT2, FOI, and anti‐PD‐L1 antibodies. Scale bar, 100 µm. M) ELISAs of IFN‐γ and TNF‐α levels in tumors from Hu‐NSG bearing PDX models treated with different combinations of SenExo‐cCCT2, FOI, and anti‐PD‐L1 antibodies (n = 5 per group). One‐way ANOVA with ​Dunnett's post‐hoc test (E–I), one‐way ANOVA with Tukey's post‐hoc test (K, M). Log rank test was used to compare the survival time. Not significant (ns); ^*^ *P* < 0.05; ^**^ *P* < 0.01; ^***^ *P* < 0.001; ^****^ *P* < 0.0001. A, J created with BioRender.com.

Based on these findings, we propose a therapeutic strategy wherein the pre‐delivery of cCCT2 into PDAC cells enhances their susceptibility to senescence post‐chemotherapy and triggers a chain reaction that ultimately inhibits PDAC progression. The introduction of circRNAs requires a delivery vehicle. Exosomes, as advantageous nanocarriers, are ideal drug carriers because of their good biocompatibility, low immunogenicity, and deformable structure.^[^ ^41^, ^42^, ^43^ ^]^ Therefore, we engineered a smart exosome system (SenExo‐cCCT2) capable of tumor targeting via iRGD and evading mononuclear phagocyte system (MPS) clearance via CD47 signaling molecules (**Figure** **8**A). The morphology and size distribution of the exosomes were verified via transmission electron microscopy and a nanolaser particle detector (Figure 8B,C). Western blotting revealed the characteristic exosome membrane proteins CD9, CD81, and TSG101, confirming the identity of SenExo‐cCCT2 (Figure 8D). We confirmed that cCCT2 was enriched in SenExo‐cCCT2 cells (Figure 8E; Figure S10B, Supporting Information). Both in vitro and in vivo experiments demonstrated that cCCT2 exhibited increased stability and a longer half‐life when protected within exosomes (Figure S10C,D, Supporting Information). Compared with normal Exo‐EVs, SenExo‐cCCT2 was taken up more by pancreatic cancer cells, reducing its clearance by macrophages (Figure 8F; Figure S10A, Supporting Information). In addition, in the in vivo drug distribution experiments, we found that more SenExo‐cCCT2 was delivered to the site where the tumor was located, with reduced capture by macrophage‐enriched organs such as the liver and spleen (Figure S10A,E, Supporting Information).

**Figure 9:**
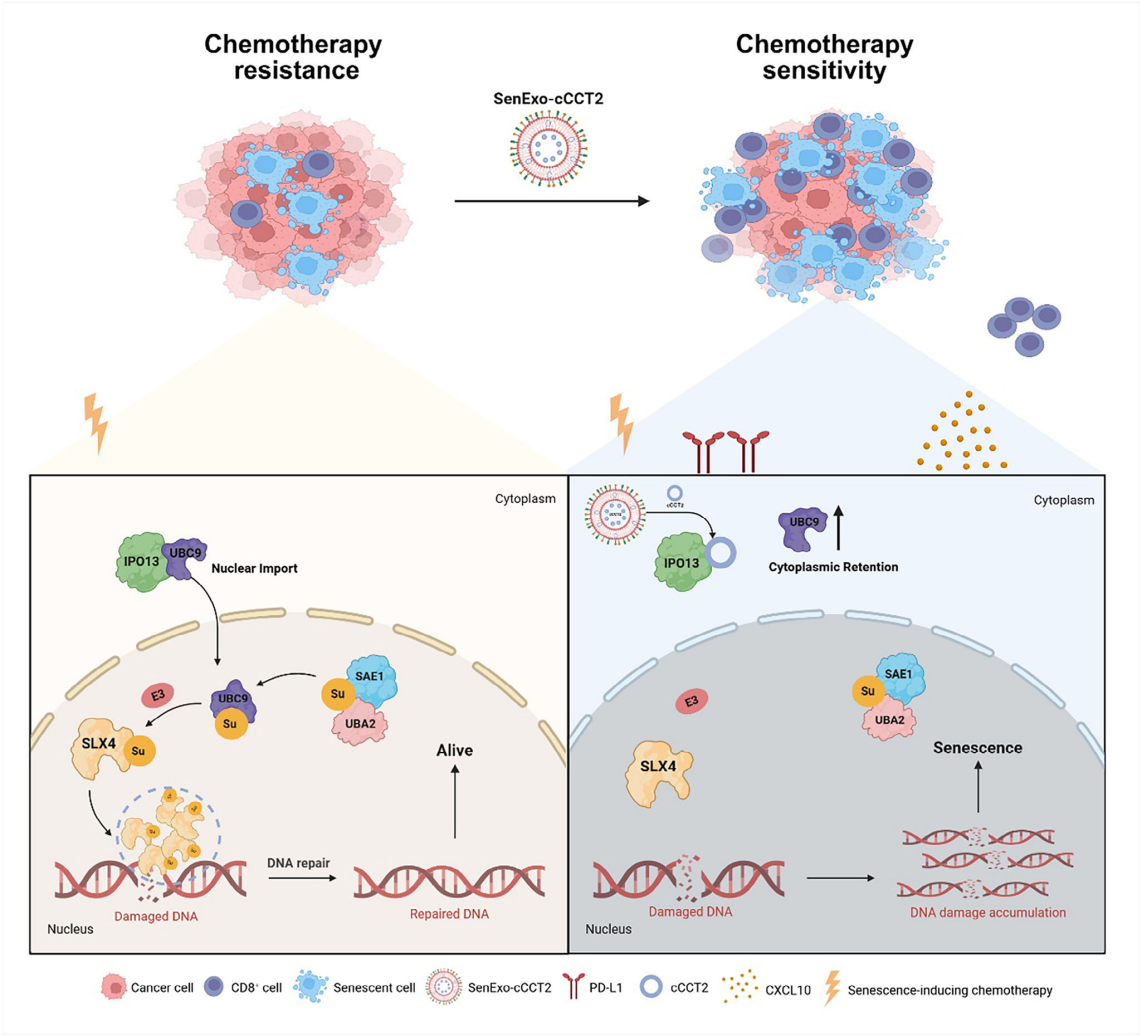
Schematic illustration of SenExo‐cCCT2 combined with chemotherapy in inducing pancreatic cancer cell senescence and modulating immune responses SenExo‐cCCT2 enables targeted delivery of cCCT2 to pancreatic cancer cells, where it disrupts IPO13‐mediated nuclear translocation of UBC9, impairing SUMOylation‐dependent DNA damage repair and inducing senescence. Senescent tumor cells secrete CXCL10 to recruit CD8⁺ T‐cells while upregulating PD‐L1. This dual mechanism enhances chemosensitivity and sets the stage for effective sequential immunotherapy. (Schematic illustration created with BioRender.com).

The ex vivo efficacy of SenExo‐cCCT2 was also evaluated. In vitro, SenExo‐cCCT2 exhibited robust senescence‐inducing effects and increased the chemosensitivity of PDAC cells with different genetic mutations; similar effects were observed in PDOs (Figure 8G–I; Figure S11A,B, Supporting Information). In the in vivo experiments, after we successfully constructed the HuNSG‐PDX model, we used SenExo‐cCCT2, chemotherapy, and an anti‐PD‐L1 antibody for sequential treatment (Figure 8J). Compared to the other groups, the triple sequential therapy group presented a significantly lower tumor growth rate and better survival time (Figure 8K). mIF confirmed the presence of more senTCs in tumor tissues treated with the triple sequential therapy, which corresponded to increased PD‐L1 expression and CD8^+^ T‐cell infiltration (Figure 8L; Figure S11C, Supporting Information). The levels of TNF‐α and IFN‐γ were significantly increased in the triple sequential therapy group (Figure 8M). In addition, no serious histopathological or serological abnormalities were observed in major organs, demonstrating the relative safety of SenExo‐cCCT2 (Figure S11D,E, Supporting Information). Furthermore, we did not observe a significant increase in the proportion of senTCs acquiring stem‐like features following senescence induction by SenExo‐cCCT2 combined with FOI (Figure S12A, Supporting Information). Notably, SenExo‐cCCT2 also exhibited potent senescence‐inducing and chemosensitizing effects when combined with the AG regimen (Figure S12B–E, Supporting Information). These data suggest that SenExo‐cCCT2 is an effective strategy for the treatment of PDAC that can increase the chemosensitivity and immunotherapy sensitivity of PDAC and that cCCT2 can be used as a novel biomarker for PDAC treatment (**Figure** **9**).

## 3 Discussion

Chemotherapy, a mainstay in pancreatic cancer treatment, is frequently complicated by acquired resistance during prolonged administration.^[^ ^44^ ^]^ In this study, a comparative analysis of senescence and apoptosis in patients receiving AG and FOLFIRINOX regimens revealed senescence as a superior biomarker of chemotherapy response compared with apoptosis, positioning senescence induction as a viable therapeutic intervention. Leveraging this insight, we identified a circRNA, cCCT2, that is highly correlated with post‐chemotherapy senescence in PDAC. Functional validation across in vitro and in vivo experimental models demonstrated that cCCT2 overexpression induces senTCs following chemotherapy, thereby augmenting chemosensitivity.

Current research on the relationship between circRNAs and senescence phenotypes in cancer remains in its infancy.^[^ ^32^, ^45^, ^46^, ^47^ ^]^ In PDAC, a study demonstrated that circHIF‐1α enhanced HIF‐1α by targeting miR‐375 and HUR, thereby delaying cellular senescence and consequently accelerating tumor growth.^[^ ^46^ ^]^ In nasopharyngeal carcinoma (NPC), circWDR37 promotes chemotherapy‐induced senescence in NPC cells,^[^ ^32^ ^]^ which is consistent with the pro‐senescent properties of cCCT2 identified in our study. However, circWDR37‐mediated senescence in NPC triggered transcription of SASP components (e.g., IL‐1α, IL‐1β, IL‐8, and CCL20), ultimately facilitating metastasis of senTCs. Conversely, circRNAs play divergent roles in other cancer contexts. Wu et al. demonstrated that the knockdown of circDNA2v antagonized tumorigenesis by potently inducing cellular senescence in colorectal cancer. SenTCs in this model preferentially secrete CXCL10 and IL‐9, thereby enhancing CD8^+^ T‐cell chemotaxis and cytolytic activity.^[^ ^45^ ^]^ Thus, circRNA‐mediated senescence phenotypes in tumor cells display significant functional heterogeneity, warranting further mechanistic investigation.

DNA damage, a central driver of cellular senescence, underpins both chemotherapy sensitivity and resistance.^[^ ^48^, ^49^ ^]^ Proteomic and cellular functional analyses revealed that cCCT2 exacerbates chemotherapy‐induced senescence by impeding the DDR process. Unlike most circRNAs that modulate single DDR pathways,^[^ ^50^ ^]^ cCCT2 broadly sensitizes PDAC cells to irinotecan, oxaliplatin, and 5‐fluorouracil, suggesting pleiotropic regulation of diverse DDR mechanisms. Mechanistically, cCCT2 targets SLX4, a scaffold protein that orchestrates multiple DDR complexes, including DPCs, ICLs, DSBs, and replication stress at vulnerable genomic loci such as common fragile sites and telomeres.^[^ ^51^, ^52^ ^]^ We hypothesize that the diverse repair functions mediated by cCCT2 are dependent on SLX4, a notion supported by our rescue experiments. We further demonstrated that cCCT2 downregulated SLX4 by competitively binding to IPO13, thereby inhibiting the nuclear import of UBC9 and impairing SLX4 SUMOylation. Previous studies have established that SUMOylated SLX4 is ubiquitinated in the nucleus by SUMO‐targeted ubiquitin ligases (STUbLs).^[^ ^26^ ^]^ Here, we unveiled a novel degradation pathway wherein cCCT2 suppresses SLX4 SUMOylation, promoting cytoplasmic export and ubiquitin‐proteasome‐dependent degradation.

Notably, our study revealed that senTCs contribute to the remodeling of the immune microenvironment. cCCT2‐driven senescence enhances CD8^+^ T‐cell infiltration, a phenomenon attributable to the SASP. Paradoxically, these infiltrating CD8^+^ T‐cells displayed diminished cytotoxic activity, which was linked mechanistically to elevated PD‐L1 expression in senTCs, consistent with recent reports of PD‐L1 as an immune evasion mechanism in senescence.^[^ ^53^, ^54^ ^]^ Building on emerging evidence that PD‐L1 blockade represents a novel senolytic strategy.^[^ ^22^, ^55^ ^]^ we employed an in vitro co‐culture system to demonstrate that anti‐PD‐L1 antibody intervention restored CD8^+^ T cell‐mediated tumor‐killing capacity. These findings provide a preclinical rationale for combining senescence induction with PD‐L1‐targeted immunotherapy to enhance the effects of chemotherapy.

Based on the above findings, we propose a novel two‐step therapeutic paradigm that integrates senescence induction with immunotherapy for pancreatic cancer. Step 1 combines cCCT2 with FOLFIRINOX chemotherapy to drive tumor cell senescence and remodel the tumor immune microenvironment. Step 2 involves sequential anti‐PD‐L1 immunotherapy to selectively eliminate senTCs and enhance chemotherapeutic efficacy. To achieve tumor‐specific cCCT2 delivery, we engineered SenExo‐cCCT2 functionalized with iRGD for active tumor targeting and CD47 to evade mononuclear phagocytic system clearance. In PDX models, a sequential triad regimen of SenExo‐cCCT2, FOLFIRINOX, and anti‐PD‐L1 demonstrated favorable treatment efficacy and safety profiles. Although SenExo‐cCCT2 has not yet advanced to clinical application, our results suggest that patients whose pathology biomarkers indicate high cCCT2 expression could be candidates for first‐line treatment with FOLFIRINOX, followed sequentially by anti‐PD‐L1 immunotherapy to achieve the desired two‐step therapeutic effect. For patients with low cCCT2 expression, our current data do not rule out the possibility of combining FOLFIRINOX with anti‐PD‐L1, but its therapeutic efficacy remains suboptimal, necessitating validation in prospective trials to identify responsive subpopulations.

This study has several limitations. First, despite balancing the molecular subtypes, PDAC heterogeneity (e.g., differentiation states and immune infiltration subtypes at various stages) may differentially modulate cCCT2‐mediated senTC effects across tumor microenvironments. Second, although the PDX models showed no increase in stemness after 6 weeks of senescence induction by Smart‐exo‐cCCT2 + FOI, this may be partly due to the relatively short duration of senTC persistence. The potential risk of senTC reactivation during prolonged induction of senescence warrants further investigation. Critically, this reactivation risk necessitates timely immunotherapy to eliminate senTCs. Third, although humanized mouse models partially reconstruct human immunity, they do not completely recapitulate the intact human immune system. Crucially, the absence of DNA sequence conservation in cCCT2 precludes its validation in syngeneic immunocompetent mouse tumor models, limiting the mechanistic exploration of these concepts.

In conclusion, the combination of senescence induction with immunotherapy is a viable strategy for chemosensitization. This study highlights cCCT2 as a predictive biomarker in chemotherapy‐responsive patients with PDAC and reveals that cCCT2 promotes the onset of tumor cell senescence by hindering the DDR process to achieve chemosensitization. A sequential regimen integrating SenExo‐cCCT2, FOLFIRINOX, and anti‐PD‐L1 antibodies suppressed pancreatic cancer progression, providing new insights into the induction of senescence in the context of conventional chemotherapy and combined immunotherapy.

## 4 Experimental Section

### Human PDAC Specimens

A total of 96 surgically resected PDAC samples with complete clinicopathological records (including gender, age, tumor dimensions, and TNM stage) were collected from Fuzhou University Affiliated Provincial Hospital. The research protocol was approved by the Ethics Committee of Fuzhou University Affiliated Provincial Hospital (Fuzhou, China). Patients or their guardians signed an informed consent form before participation in the study. Chemotherapy sensitivity and resistance were defined by radiological assessment in accordance with the RECIST 1.1 criteria.^[^ ^56^ ^]^ Patients were deemed neoadjuvant chemotherapy‐sensitive (NAC‐S) if imaging demonstrated tumor shrinkage (complete response [CR] or partial response [PR]), whereas neoadjuvant chemotherapy‐resistant (NAC‐R) was assigned to those showing tumor enlargement (progressive disease [PD]) or the appearance of new lesions. The classification into “senescence‐high” and “senescence‐low” groups was based on the median proportion of SA‐β‐gal positive cells observed in tissue sections, with values above this threshold designated as “senescence‐high” and those below as “senescence‐low”.

### Single‐Cell Sequencing and Data Analysis

Three pairs of pancreatic cancer tissue samples from NAC‐R and NAC‐S patients were collected. Single‐cell isolation and mRNA capture were performed using the Singleron Matrix system (Singleron, China). Subsequently, scRNA‐seq libraries were constructed with the GEXSCOPE Single‐Cell RNA Library Kit (Singleron, China) through reverse transcription, amplification, and library preparation. Only libraries meeting quality standards were sequenced on the Illumina HiSeq 6000 platform (150 bp paired‐end reads). Raw single‐cell RNA sequencing data were processed using the CeleScope pipeline (v1.12.0) with default parameters. UMI count matrices derived from four single‐cell libraries were merged and filtered in R (v4.3.1) with Seurat (v5.1.0) under the following criteria: genes detected in fewer than three cells, cells with fewer than 500 detected genes, and cells where >25% of UMI counts mapped to mitochondrial genes. Doublets were removed using “scDblFinder”.^[^ ^57^ ^]^ Gene expression data were normalized via the LogNormalize method using the NormalizeData function. The top 2000 variable genes were identified with FindVariableFeatures, followed by data scaling (ScaleData) and principal component analysis (PCA). To enhance integration across the samples, the “Harmony” was applied. t‐SNE or UMAP dimensionality reduction and cell clustering were performed using Seurat's FindNeighbors and FindClusters. Cell types were annotated based on canonical markers reported in the literature.^[^ ^58^ ^]^ Tumor cell and T‐cell clusters were extracted for further sub‐clustering and analysis. Expression of senescence markers was visualised using “Nebulosa”,^[^ ^59^ ^]^ and senescence‐related gene set enrichment scores were calculated via “GSVA”.^[^ ^60^ ^]^ CDKN1A, CDKN2A, and CDKN2B were used as the markers of senTC, and senescence‐related pathways were utilized for validation.^[^ ^33^, ^34^, ^35^ ^]^

### circRNA‐Seq Analysis

circRNA‐seq analysis was performed on three paired samples grouped by senescence level (high vs low) and three paired surgical samples classified as NAC‐R and NAC‐S groups. Total RNA was extracted with TRIzol reagent (Invitrogen, CA, USA). Strand‐specific RNA sequencing libraries were prepared from total RNA using the KC‐Digital Chain mRNA Library Preparation Kit and Illumina‐compatible circRNA libraries (SeqHealth Technology, Wuhan, China) according to the manufacturer's instructions. Pre‐amplified cDNA molecules were labeled with unique molecular identifiers (UMIs) containing eight random bases to eliminate PCR and sequencing biases. Libraries corresponding to 200–500 bp fragments were enriched, quantified, and sequenced on the DNBSEQ‐T7 platform (MGI Tech, Shenzhen, China) using a PE150 (paired‐end 150 bp) model.

### Proteomic Analysis

FOI‐induced senescent Capan‐1 cells with either EV or overexpressing cCCT2 were lysed in buffer containing 2% SDS, 7 m urea, and 1 mg mL^−1^ protease inhibitor cocktail. Cell lysates were homogenized on ice for 3 min using an ultrasonic homogenizer, then centrifuged at 15 000 rpm for 15 min at 4 °C to collect the supernatant. Proteins were digested with sequencing‐grade modified trypsin (Promega, Madison, WI, USA) at 37 °C for 16 h at a ratio of 50:1 (w/w). Following the manufacturer's instructions with minor modifications, peptides were labeled using the iTRAQ labeling kit (Applied Biosystems). The labeled peptide mixtures were subsequently dried, reconstituted, and fractionated using strong cation exchange (SCX) chromatography on an LC‐20AB HPLC system (Shimadzu, Japan).Online nano‐electrospray LC‐MS/MS analysis was carried out using an Orbitrap Fusion Lumos mass spectrometer coupled with an EASY‐nLC 1200 system (Thermo Fisher Scientific, MA, USA), employing a C18 analytical reverse‐phase column (operated at 40 °C with a flow rate of 200 nL min^−1^). The mass spectrometer was operated in data‐independent acquisition (DIA) mode, alternating automatically between MS and MS/MS scans. Raw DIA data were processed and analyzed using Spectronaut X (Biognosys AG, Switzerland) with default settings. All experimental procedures and data analysis were conducted by Gene Denovo Biotechnology Co., Ltd. (Guangzhou, China). Differentially expressed proteins (DEPs) were identified based on Student's t‐test, with a false discovery rate (FDR) Q‐value < 0.05 and an absolute log2 fold change > 0.58 considered significant. Heatmaps of significant DEPs between the cCCT2‐overexpressing and control groups were visualized. Gene Set Enrichment Analysis (GSEA) was subsequently performed to explore relevant pathways.

### Genomic Analysis of PDAC Tissues

Genomic profiling was performed on fresh tumor tissues obtained from PDAC patients following surgical resection using the Oseq platform (BGI Genomics, Shenzhen, China), a clinically validated assay designed to detect single nucleotide variants (SNVs), small insertions and deletions (indels), copy number alterations, and gene fusions across a panel of 688 cancer‐related genes. In accordance with the manufacturer's protocol (Integrated DNA Technologies, Coralville, IA, USA), 400 ng of DNA extracted from fresh tumor tissue was used to construct barcoded libraries. Target enrichment of both exonic and intronic regions was carried out using custom‐designed probes from IDT. Sequencing was performed on the MGISEQ‐2000 platform (MGI, Shenzhen, China). Genomic alterations were identified using the associated analysis pipeline. Data interpretation focused on clinically actionable alterations, based on guidelines from the National Comprehensive Cancer Network (NCCN), the Association for Molecular Pathology (AMP), the American Society of Clinical Oncology (ASCO), and the College of American Pathologists (CAP).

### Cell Culture and Drug Treatment

The human PDAC cell lines PANC‐1 (RRID: CVCL_0480), BxPC‐3 (RRID: CVCL_0186), Capan‐1 (RRID: CVCL_0237), MIA PaCa‐2 (RRID: CVCL_0428), AsPC‐1 (RRID: CVCL_0152) and SW1990 (RRID: CVCL_1723) were procured from the American Type Culture Collection (ATCC; Manassas, VA, USA). Cells were cultured in standard medium recommended by ATCC, supplemented with 10% heat‐inactivated fetal bovine serum (FBS) and antibiotics (50 U mL^−1^ penicillin and 50 mg L^−1^ streptomycin), under a humidified atmosphere of 5% CO₂ at 37 °C. Mycoplasma contamination was routinely excluded using the Universal Mycoplasma Detection Kit.

Based on the measured IC_25_ values, the concentrations of the FOI drug combination were determined. In non‐senescence‐induced functional cellular experiments, Capan‐1 cells were treated with the FOI combination (Fluorouracil: 4 µm, Oxaliplatin: 3 µm, SN38: 15 nm) for 48 h, while MIA PaCa‐2 cells were treated with a modified FOI regimen (Fluorouracil: 3 µm, Oxaliplatin: 1 µm, SN38: 10 nm) for the same duration.

### Senescent Cell Induction

Based on the determined IC_25_ values, one‐fifth of the IC_25_ concentration was selected as the senescence‐inducing dose for the cells. Capan‐1 cells were treated with the FOI combination (fluorouracil: 0.8 µm, oxaliplatin: 0.6 µm, SN38: 3 nm) for one week. MIA PaCa‐2 cells were treated with the FOI combination (fluorouracil: 0.6 µm, oxaliplatin: 0.2 µm, SN38: 2 nm) for one week. Subsequently, morphological changes were observed, and SA‐β‐gal staining was performed to assess cellular senescence.

### Cell Transfection

The hsa_circ_0002940 (cCCT2) lentivirus and plasmids were obtained from GeneChem (Shanghai, China). Lentiviral transduction was performed by GeneChem (Shanghai, China), while plasmid transfection was carried out using Roche transfection reagent (Roche, 578 Basel, Switzerland).

### Patient‐Derived Organoid (PDO) Culture

Tumor specimens from patients with PDAC were finely disaggregated in Advanced Dulbecco's Modified Eagle Medium/F12 (Gibco, CA, USA) containing Enzymatic Dissociation Solutions I and II (STEMCELL, CA, USA). The enzymatic reaction was quenched by adding Advanced DMEM/F12 enriched with 10% fetal bovine serum. The cell pellet was then reconstituted in Matrigel (Corning, NY, USA), dispensed into 24‐well plates and allowed to gel at 37 °C for 30 min. Thereafter, organoids were maintained at 37 °C in a humidified atmosphere with 5% CO₂, with culture medium refreshed twice weekly. Organoid sizes were randomly recorded under optical microscopy at 100 × magnification.

### RNA Isolation and RT‐qPCR Analysis

Total RNA was extracted with TRIzol Reagent (Thermo Fisher Scientific, USA) according to the manufacturer's instructions. First‐strand cDNA was synthesized using the PrimeScript RT Kit (TaKaRa, Shanghai, China). Quantitative real‐time PCR was performed on a StepOnePlus system (Thermo Fisher Scientific, USA), and transcript levels of mRNA and circRNA were normalized to glyceraldehyde‐3‐phosphate dehydrogenase (GAPDH) using the 2^−ΔΔCT^ method.

### Ribonuclease R (RNase R) Treatment

To distinguish circular from linear RNA, total RNA was incubated with or without 3 U µg^−1^ RNase R (Genseed, Guangzhou, China) at 37 °C for 30 min. Following treatment, the abundance of cCCT2 and linear CCT2 transcripts was determined by RT‐qPCR.

### Actinomycin D Assay

Cells were exposed to actinomycin D (Sigma‐Aldrich) at 2 µg mL^−1^ for specified durations. At each time point, RNA was extracted and RT‐qPCR was performed to assess the decay kinetics of the transcripts.

### Agarose Gel Electrophoresis

RNA samples were resolved on 2% (w/v) agarose gels in 1 × TAE buffer at 120 V for 30 min. Gels were imaged using the ChemiDoc MP System (Bio‐Rad, CA, USA).

### Fluorescence In Situ Hybridization (FISH)

A cCCT2‐specific fluorescent probe was designed and synthesized by Servicebio (Wuhan, China). Cells were fixed, permeabilized and pre‐hybridized before overnight hybridization with the probe at 37 °C. Post hybridization, samples were washed sequentially in 4×, 2× and 1 × SSC containing 0.1% Tween‐20 at 42 °C. Nuclei were counterstained with DAPI, and images were captured on a confocal microscope (Servicebio, Wuhan, China).

### Senescence‐Associated β‐Galactosidase (SA‐β‐gal) Staining

SA‐β‐gal staining was performed using a commercial SA‐β‐gal staining kit (Beyotime Biotechnology, Shanghai, China), following the manufacturer's protocol. For quantification, SA‐β‐gal‐positive cells were observed and counted under a light microscope.

### Cell Viability Assay

Cell growth was assessed using the Cell Counting Kit‐8 (CCK‐8; Dojindo, Kumamoto, Japan). Absorbance was measured at 450 nm with a microplate reader (Tecan Trading AG, Switzerland). Raw readings were first corrected by subtracting the absorbance of blank wells, and cell viability was then normalized to that of wells treated with vehicle (DMSO). Following 48 h of exposure to the indicated treatments, the concentrations resulting in 25% and 50% inhibition (IC₅₀ and IC₂₅) were determined.

### Live/Dead Staining of 3D Tumor Spheroids

Tumor cells were washed three times with PBS and dissociated into a single‐cell suspension using a cell dissociation enzyme. The cells were transferred to a sterile vessel, then distributed (1 500 cells per well) into ultra‐low‐attachment 96‐well round‐bottom plates. Spheroids were allowed to form over 72 h at 37 °C in a humidified 5% CO₂ atmosphere. After 48 h’ exposure to FOI, spheroids were stained with the Live/Dead Cell Imaging Kit (calcein‐AM/PI; BestBio, Shanghai, China) according to the supplier's protocol, and images were captured by inverted fluorescence microscopy.

### Western Blotting

Proteins were extracted from PDAC tissues or cell lines with RIPA lysis buffer (Solarbio, Beijing, China). Nuclear and cytoplasmic fractions were separated using a dedicated kit (Thermo Fisher Scientific, MA, USA). Protein concentrations were determined by BCA assay (Beyotime, Beijing, China). Equal amounts of protein were resolved by SDS–PAGE, transferred to PVDF membranes, then blocked in 5% non‐fat milk for 1 h. Membranes were probed overnight at 4 °C with primary antibodies, washed, then incubated with HRP‐conjugated secondary antibodies. Bands were visualised using Pierce ECL substrate (Thermo Fisher Scientific, MA, USA).

### Comet Assay

The comet assay was carried out using the Trevigen Comet Assay Kit, following the manufacturer's instructions. Briefly, the cells were embedded in low‐melting‐point agarose on comet slides and lysed overnight. The following day, slides were subjected to electrophoresis in neutral unwinding buffer at 25 V for 30 min. After electrophoresis, the gels were neutralized and stained with SYBR Gold (Invitrogen, Carlsbad, CA, USA). Fluorescent images were captured using a fluorescence microscope, and tail moments were analyzed using dedicated comet scoring software.

### CD8^+^ T‐Cell Isolation and Ativation

Peripheral blood mononuclear cells (PBMCs) were isolated from human donors by density gradient centrifugation over Ficoll (Fcmacs, Nanjing, China). CD8⁺ T‐cells were purified by negative selection using a magnetic kit (Miltenyi Biotec, Bergisch Gladbach, Germany). Freshly isolated CD8⁺ cells were cultured in plates pre‐coated with 3.5 µg mL^−1^ anti‐CD3 and 1 µg mL^−1^ anti‐CD28 antibodies (BioLegend, San Diego, CA, USA) in RPMI‐1640 supplemented with 10% FBS, 1% antibiotics and 10 ng mL^−1^ recombinant human IL‐2 (PeproTech, Rocky Hill, NJ, USA). Cultures were maintained for 5–7 days, with fresh IL‐2‐containing medium added every 48 h.

### CD8^+^ T‐Cell Migration Assay

Activated CD8⁺ T‐cells (5 × 10⁶ cells/well) were placed in the upper chamber of a Transwell insert (5 µm pore; Corning). Conditioned medium of senTCs with EV or overexpressing cCCT2 was added to the lower chamber as chemoattractant. After 6 h at 37 °C, migrated T‐cells in the lower chamber were collected and counted.

### CD8^+^ T‐Cell‐Mediated Tumor Cell Killing Assay

The senTCs with EV or overexpressing cCCT2 (5  × 10⁴ cells/well) were seeded in 24‐well plates. Activated CD8⁺ T‐cells were added at an effector‐to‐target ratio of 10:1 and co‐cultured in complete DMEM for 48 h. Supernatants were harvested and centrifuged at 400  × g for 5 min, then assayed for lactate dehydrogenase (LDH) release using a commercial kit (Beyotime) per the manufacturer's instructions. Absorbance was read at 490 nm.

### Enzyme‐Linked Immunosorbent Assay (ELISA)

Levels of TNF‐α and IFN‐γ in co‐culture supernatants were quantified using human TNF‐α and IFN‐γ ELISA kits (Shanghai Enzyme‐linked Biotechnology Co., Ltd.) following the manufacturer's protocols.

### Immunohistochemistry (IHC) and Hematoxylin and eosin (HE) Staining

Tumor specimens were fixed in 4% paraformaldehyde, paraffin‐embedded, sectioned, then deparaffinized and rehydrated. Antigen retrieval was performed in citrate buffer using heat induction. Sections were blocked in 5% BSA for one hour, incubated with primary antibody overnight at 4 °C, then with HRP‐conjugated secondary antibody for one hour at room temperature. Staining was developed with DAB, and slides were counterstained with hematoxylin.

### Multiplex Immunofluorescence Staining and Image Analysis

Frozen pancreatic cancer tissue microarray sections were co‐stained for Pan‐CK, caspase‐3, and CD8 using a multiplex fluorescence kit (RecordBio Biological Technology, Shanghai, China), supplemented with SPiDER‐β‐Gal (DOJINDO, Tokyo, Japan) staining to visualize cellular senescence distribution, following the manufacturer's protocol. Fluorescent images were acquired using the Vectra 2 Automated Slide Analysis System with Vectra software v2.0.8 (PerkinElmer). Caspase‐3 was used as an indicator of apoptosis^[^ ^61^ ^]^ and SPiDER‐β‐Gal (SA‐β‐Gal) was used to indicate senescence,^[^ ^62^ ^]^ and calculated the proportion of positive cells for each marker. The senescence‐to‐apoptosis ratio (SAR) was calculated as: SAR = senescent positive cells/(senescent positive cells + apoptotic positive cells), to assess the relative contributions of senescence and apoptosis to therapeutic response.

### RNA Antisense Purification (RAP) Assay

RAP was carried out using the RNA Antisense Purification Kit (BersinBio, Guangzhou, China) according to the manufacturer's instructions. Co‐purified proteins were visualized by silver staining (Solarbio, Beijing, China), and associated RNAs were analyzed by RT‐qPCR.

### RNA Immunoprecipitation (RIP) Assay

RIP was performed with the Magna RIP RNA‐Binding Protein Immunoprecipitation Kit (Merck Millipore, MA, USA) following the supplier's protocol. Co‐precipitated RNAs were extracted and quantified by RT‐qPCR.

### Exosome Preparation

In this study, 293T/293F cells were utilized as donor cells for exosome production. Cells were transfected with cCCT2 and CD47 overexpression lentiviral vector. These cells were then cultured in 3D conditions using serum‐free media to augment exosome secretion and production. Upon verification of stable expression, exosomes were isolated from the conditioned media. Initially, culture supernatants were centrifuged at 2000 × g for 10 min, followed by 10 000 × g for 30 min to eliminate cells and debris. The resulting supernatants were filtered through a 0.22 µm membrane to remove large particles and subsequently ultracentrifuged at 100 000 × g for 70 min. The pellet was washed in PBS to remove contaminating proteins, and the ultracentrifugation step was repeated. To load iRGD peptides onto the exosomes, the iRGD lipid‐anchor labeling kit was employed. After labeling, exosomes were washed, concentrated, resuspended in PBS, and stored at −80 °C until use.

### Exosome Characterization

The morphology of the isolated exosomes was examined using transmission electron microscopy (TEM). Briefly, exosomes were deposited onto copper grids, stained with 2% uranyl acetate, air‐dried for 30 min, and imaged using a JEM‐2000 EX TEM (JEOL Ltd., Tokyo, Japan). For particle size distribution analysis, exosomes were diluted to a protein concentration of 500 ng mL^−1^ and analyzed using a NanoSight NS300 nanoparticle tracking analysis system (Malvern Instruments Ltd., UK). According to previous reports,^[^ ^63^ ^]^ the abundance of cCCT2 in SenExo‐cCCT2 was evaluated by determining the copy number of circRNA using absolute RNA quantification. Briefly, standard curve was created by series dilution of purified and quantified PCR products of indicated circRNAs. The copy number of cCCT2 was then quantified based on this standard curve and normalized to the number of exosome particles determined by nanoparticle tracking analysis (NTA).

### In Vivo Exosome Biodistribution

For biodistribution studies, exosomes were labeled with DiR or DiI (Invitrogen, USA) at a final concentration of 8 µm. Unbound dye was removed, and the labeled extracellular vesicles were injected intravenously into mice. After 6 h, major organs were harvested, and exosome localization was visualized using the IVIS Lumina II In Vivo Imaging System (PerkinElmer, Thermo Fisher Scientific, USA).

### Subcutaneous Xenograft Model

PDAC cells (2 × 10⁶ cells suspended in a 1:1 solution of PBS and Matrigel; final volume 100 µL per mouse) were subcutaneously injected into the dorsum of nude mice (Shanghai Model Organisms, China). Seven days after implantation, once tumors were palpable, mice received intraperitoneal administrations of oxaliplatin (3 mg kg^−1^), 5‐fluorouracil (25 mg kg^−1^) and irinotecan (25 mg kg^−1^) once weekly for three consecutive weeks. Tumor dimensions were recorded with digital callipers, and volumes calculated accordingly. Six weeks post‐treatment initiation, all animals were humanely euthanized.

### Humanized Mouse Model of the Immune System

For the Hu‐PBMC model, peripheral blood mononuclear cells (PBMCs) were isolated from human donors using Ficoll (Fcmacs Biotech, Nanjing, China) in accordance with the manufacturer's protocol. A total of 5 × 10^6^ − 1 × 10^7^ human PBMCs were intravenously transplanted into 6‐week‐old NSG mice (NOD scid gamma; Shanghai Model Organisms, China). Reconstitution of hCD45⁺ hCD3⁺ cells in murine peripheral blood was monitored at weeks 2, 3, 4, and 5. The Hu‐PBMC model was considered successful when hCD45⁺ hCD3⁺ cells exceeded 25% in peripheral blood after two weeks.

### Humanized Cell Line‐Derived Xenograft (Hu‐CDX) Mouse Model

To establish the Hu‐CDX model, 1 × 10⁵ PDAC cells (EV, OE‐cCCT2, sh‐NC, or sh‐cCCT2) in the logarithmic growth phase were suspended in a 1:1 solution of PBS and Matrigel and orthotopically injected into the pancreas of Hu‐NSG mice. One week post‐tumor inoculation, intraperitoneal administration of oxaliplatin (3 mg kg^−1^), fluorouracil (25 mg kg^−1^) and irinotecan (25 mg kg^−1^) was initiated weekly for three consecutive weeks. Six weeks post‐treatment initiation, Hu‐CDX mice were euthanized, tumors excised, weighed, photographed, and subjected to multiplex immunofluorescence analysis.

### Humanized Patient‐Derived Xenograft (Hu‐PDX) Mouse Model

To establish the Hu‐PDX model, tumor specimens were used from five PDAC patients. The fresh PDAC specimens were passaged three times into new recipient mice. At the third passage, tumors were diced into ≈10 mm^3^ fragments and implanted subcutaneously into the flanks of Hu‐NSG mice. No mixing of tumor tissues occurred during passaging or re‐implantation. Each mouse was individually labeled with an ear tag and could be precisely traced back to its corresponding patient. Three weeks post‐implantation, the Hu‐PDX models from each of five independent patient‐derived tumors were randomly and evenly assigned to each treatment group, and received once‐weekly intraperitoneal doses of SenExo‐cCCT2 (30 µg), oxaliplatin (3 mg kg^−1^), fluorouracil (25 mg kg^−1^) and irinotecan (25 mg kg^−1^). On the 3rd and 6th day of each treatment week, 200 µg anti‐PD‐L1 antibody was administered intravenously. Treatments were continued for three consecutive weeks. Six weeks post‐treatment initiation, Hu‐PDX mice were euthanized, tumors excised, weighed, photographed and processed for downstream analyzes.

### Statistical Analysis

Statistical analyzes were performed using GraphPad Prism v9.0 and SPSS 23.0. Unless otherwise stated, the data are expressed as mean± standard deviation (SD). The sample size (n) of each statistical analysis is mentioned in the legend of the corresponding figure. Comparisons between two groups were made by Student's t‐test or the Mann–Whitney U test, as appropriate, while multiple groups were compared by analysis of variance (ANOVA). Overall survival (OS) and Recurrence‐free survival (RFS) were estimated by Kaplan–Meier analysis and differences assessed with the log‐rank test. χ^2^ test or Fisher's exact test were used to analyze categorical variables. A *P* value less than 0.05 was considered statistically significant. Significance levels are denoted as: ^*^ *P* < 0.05; ^**^ *P* < 0.01; ^***^ *P* < 0.001; ^****^ *P* < 0.0001; ns, not significant (*P* ≥ 0.05).

## Ethical Approval Statement

All clinical tissue samples were obtained from the Hepatobiliary and Pancreatic Surgery Department of the Fuzhou University Affiliated Provincial Hospital (Fujian, China). This study was approved by the Ethics Committee of the Fuzhou University Affiliated Provincial Hospital and conducted in accordance with its guidelines. All animal experimental protocols were reviewed and authorized by the Institutional Animal Care and Use Committee (IACUC) of Fujian Provincial Hospital (Approval No. IACUC‐FPH‐SL‐20240227[0092]), complying with the Guide for the Care and Use of Laboratory Animals.

## Conflict of Interest

The authors declare no conflict of interest.

## Author Contributions

S.Z., Y.C., H.L., and J.L. contributed equally to this work. S.Z. and S.C. designed the study. S.Z., Y.C., and H.L. performed the study and wrote the paper. S.Z., H.L., Y.C., and J.L. conducted the experiments. J.L., G.L., Y.W., and H.Z. participated in data analysis. C.L., H.C., L.J., M.H., and L.H. assisted with experiments. Y.C. and X.H. collected the tissues. Y.T., Z.W., Q.L., and S.C. provided technical help. All authors read and approved the final manuscript.

## Supporting information



Supporting Information

Supporting Information

## Data Availability

The data that support the findings of this study are available from the corresponding author upon reasonable request.
