# Supplementary material for: SenExo‐cCCT2 Reprograms Senescence Response and Anti‐Tumor Immunity Following FOLFIRINOX Chemotherapy in Pancreatic Ductal Adenocarcinoma
Source: Adv Sci (Weinh). 2025 Jul 21;12(38):e08431. doi: 10.1002/advs.202508431 (PMC12520514; doi:10.1002/advs.202508431)
Supplement: Supplementary file 1 — Supporting Information [file ADVS-12-e08431-s001.docx]

**Supplementary Figure legends**

**
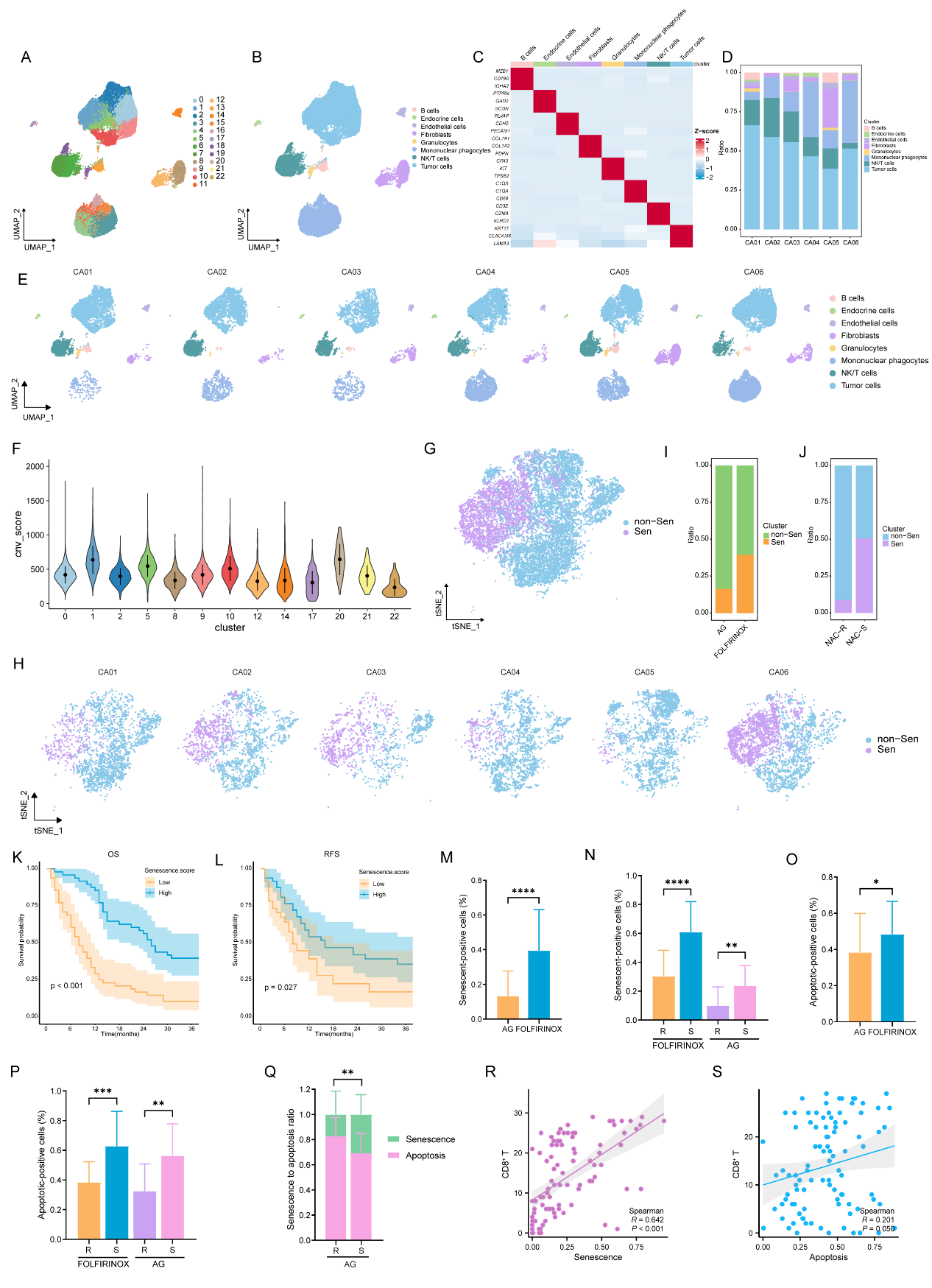
Supplementary Figure S1**

(A) UMAP plots of the cell clusters identified in human PDAC scRNA‑seq data.

(B) UMAP plots showing the distribution of annotated subclusters.

(C) Heat map of gene expression for marker genes of each subcluster.

(D) Proportions of the identified clusters in each sample.

(E) UMAP plots of subcluster distributions for each sample.

(F) Violin plots of copy number variation (CNV) for each subcluster, calculated by inferCNV.

(G) t‑SNE plots highlighting senescent tumor cells (senTCs).

(H) t‑SNE plots of senescent tumor cells subcluster distributions for each sample.

(I) Proportion of senTCs stratified by chemotherapy regimen.

(J) Proportion of senTCs stratified by the neoadjuvant chemotherapy-sensitive (NAC-R) and neoadjuvant chemotherapy-resistant (NAC-S) groups.

(K) Kaplan-Meier survival curves of overall survival (OS) for PDAC patients with different senescence levels (n = 96).

(L) Kaplan-Meier survival curves of recurrence‑free survival (RFS) for PDAC patients with different senescence levels (n = 96).

(M) Comparison of senescent-positive cells across different chemotherapy regimens (n = 96).

(N) Subgroup comparison of senescent-positive cells by response status within each chemotherapy regimen (n = 96).

(O) Comparison of apoptotic-positive cells across chemotherapy regimens (n = 96).

(P) Subgroup comparison of apoptotic-positive cells by response status within each chemotherapy regimen (n = 96).

(Q) Comparison of senescence-apoptosis ratio (SAR) by response status within the AG regimen.

(R) Correlation between senescence levels and CD8⁺ T‑cell infiltration in PDAC patients from Fuzhou University Affiliated Provincial Hospital (n = 96).

(S) Correlation between apoptosis levels and CD8⁺ T‑cell infiltration in PDAC patients from Fuzhou University Affiliated Provincial Hospital (n = 96).

The data are expressed as the means ± SDs. Unpaired two-tailed Student’s t-test or Mann-Whitney *U*-test (M-Q), log rank test (K, L), spearman anlysis (R, S). Not significant (ns); **P* < 0.05; ***P* < 0.01; ****P* < 0.001; *****P* < 0.0001.

**
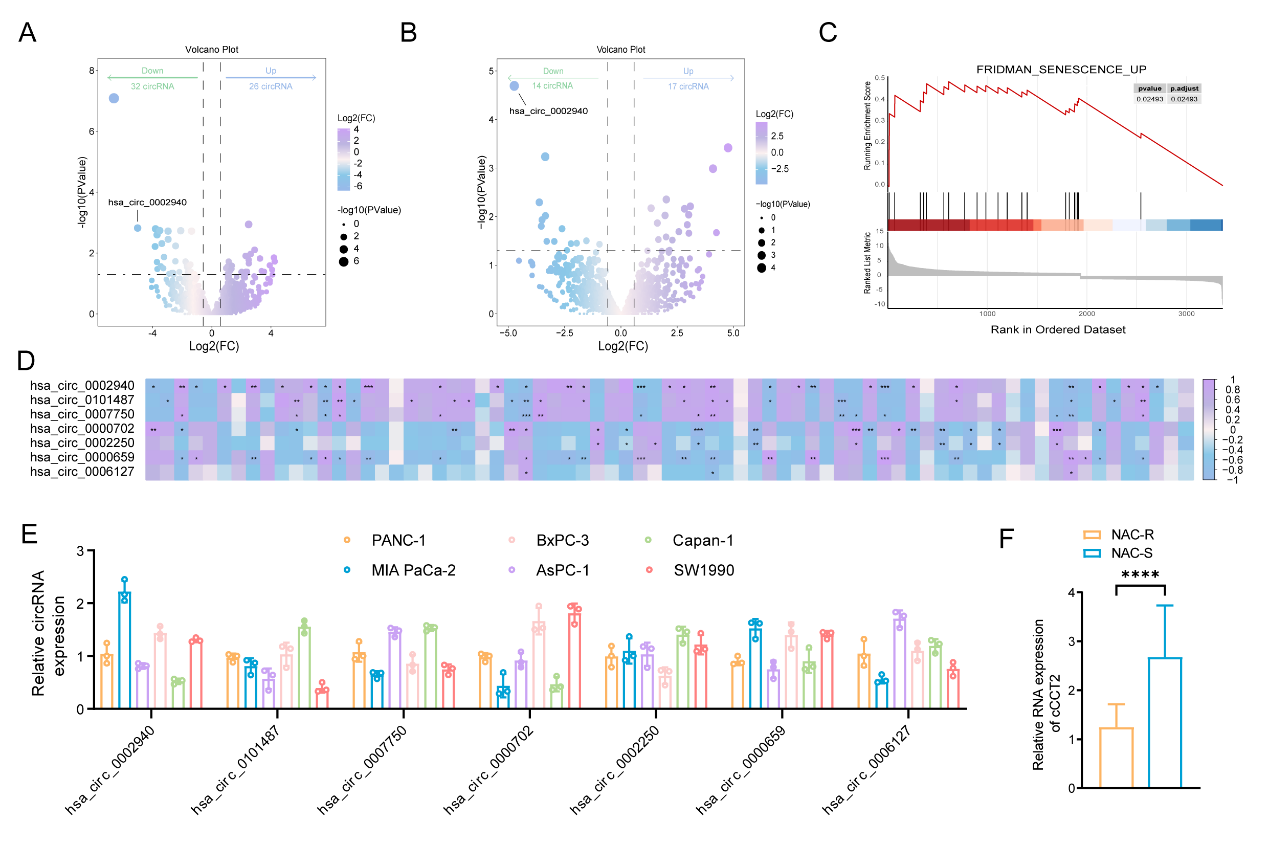
**

**Supplementary Figure S2**

(A) Volcano plot of differentially expressed circRNAs in tumors from three patient pairs with high versus low senescence. Dot color indicates normalised protein expression, dot size represents P value.

(B) Volcano plot of differentially expressed circRNAs in tumors from three patient pairs defined as chemotherapy sensitive versus resistant. Dot color indicates normalised protein expression, dot size represents P value.

(C) GSEA plot showing enrichment of the Fridman senescence pathway in high‑ versus low‑senescence groups.

(D) Correlation heat map between candidate circRNAs and senescence‑related genes.

(E) Expression levels of candidate circRNAs across common PDAC cell lines (n = 3 per group).

(F) Expression of has_circ_0002940 in PDAC patients from Fuzhou University Affiliated Provincial Hospital, stratified by chemotherapy response (n = 96).

The data are expressed as the means ± SDs. Unpaired two-tailed Student’s t-test or Mann-Whitney *U*-test (F), one-way ANOVA with Tukey’s post-hoc test (E). Not significant (ns); **P* < 0.05; ***P* < 0.01; ****P* < 0.001; *****P* < 0.0001.


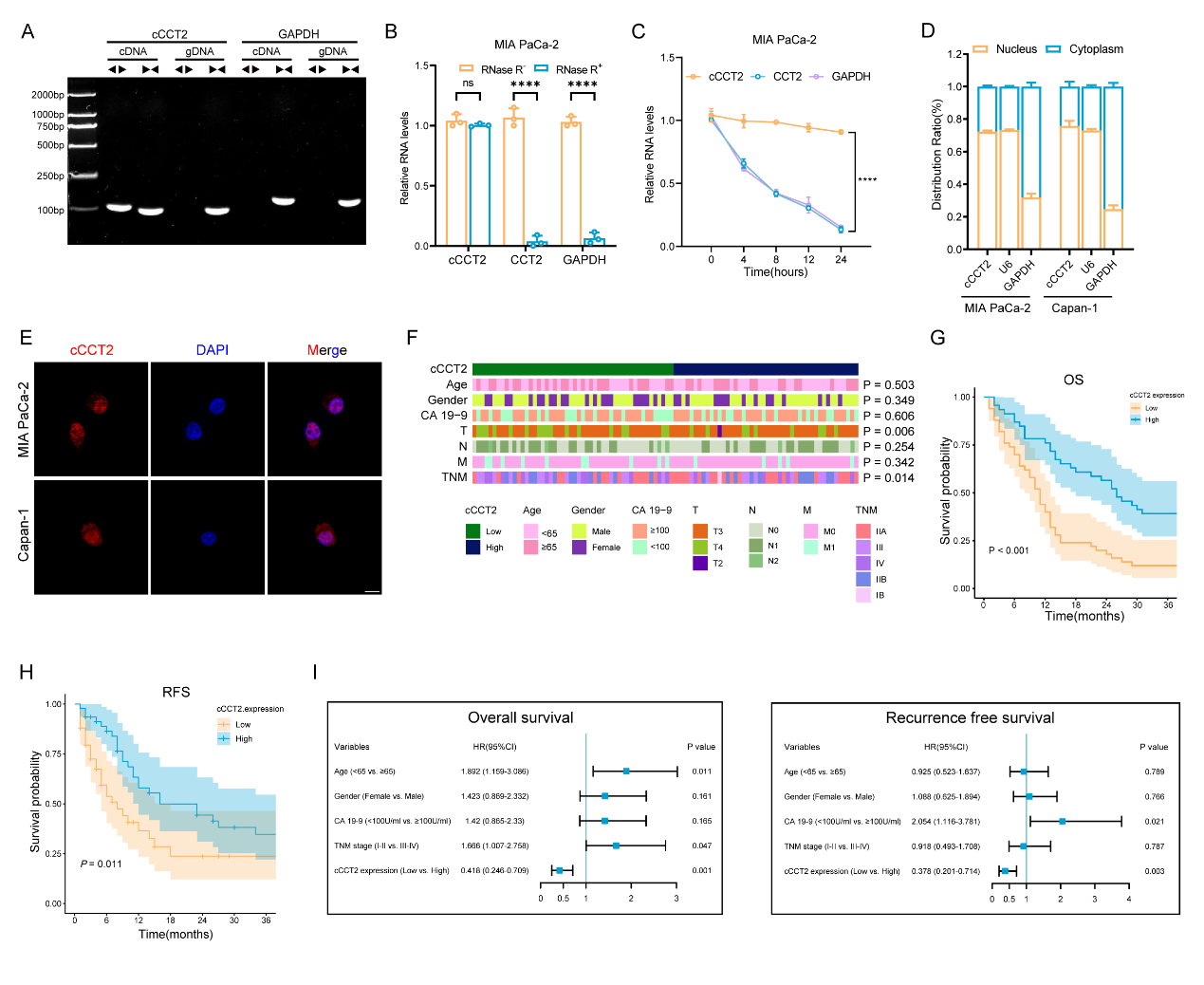


**Supplementary Figure S3**

(A) Agarose gel electrophoresis of cCCT2 amplification from cDNA and gDNA using convergent and divergent primers. GAPDH serves as negative control.

(B) RNA stability analysis of cCCT2, CCT2, and GAPDH in RNase R-treated MIA PaCa‑2 cells (n = 3 per group). GAPDH mRNA was used as a negative control.

(C) RT-qPCR analysis of cCCT2 and CCT2 stability in MIA PaCa‑2 cells following actinomycin D treatment (n = 3 per group). GAPDH mRNA serves as negative control.

(D) The intracellular localisation of cCCT2 was detected via an RNA cytoplasmic/nuclear hierarchical separation assay in MIA PaCa-2 and Capan-1 cells (n = 3 per group). U6 was used as a nuclear internal reference, and GAPDH was used as a cytoplasmic internal reference.

(E) Representative images of FISH showing nuclear and cytoplasmic distribution of cCCT2 (red); nuclei counterstained with DAPI (blue). Scale bar, 20 µm.

(F) Heat map of clinical and pathological data for high‑ versus low‑cCCT2 expression subgroups in the Fuzhou University Affiliated Provincial Hospital cohort (n = 96).

(G) Kaplan-Meier survival analysis of OS for PDAC patients with high or low cCCT2 expression (n = 96).

(H) Kaplan-Meier survival analysis of RFS for PDAC patients with high or low cCCT2 expression (n = 96).

(I) Multivariate analysis of OS and RFS in PDAC patients stratified according to cCCT2 expression (n = 96). Dots in Forest plots indicate risk ratios from Cox proportional risk models, and error bars indicate two-sided 95% confidence intervals.

The data are expressed as the means ± SDs. Unpaired two-tailed Student’s t-test or Mann-Whitney *U*-test (B), repeated measures ANOVA test (C), log rank test (G, H). Not significant (ns); **P* < 0.05; ***P* < 0.01; ****P* < 0.001; *****P* < 0.0001.


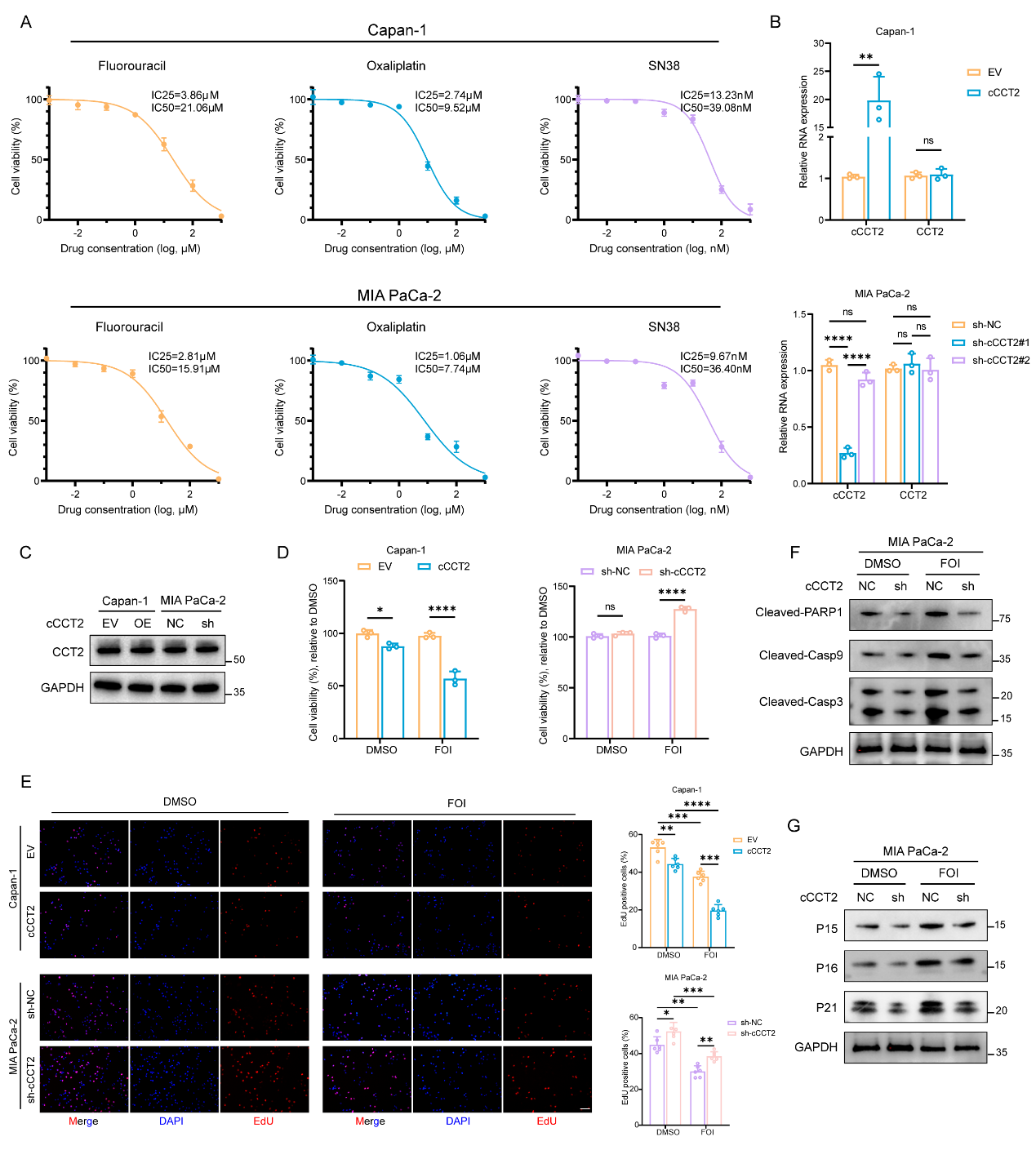


**Supplementary Figure S4**

(A) IC₂₅ and IC₅₀ values for fluorouracil, oxaliplatin and SN38 in Capan‑1 and MIA PaCa‑2 cell lines (n = 3 per group).

(B) RT-qPCR validation of cCCT2 overexpression and knockdown efficiency and effects on CCT2 mRNA levels (n = 3 per group).

(C) Western blot analysis of CCT2 protein expression in Capan‑1 and MIA PaCa‑2 cell lines with cCCT2 overexpression or knockdown.

(D) Cell viability assays in Capan‑1 and MIA PaCa‑2 cell lines with cCCT2 overexpression or knockdown treated with the FOI combination (fluorouracil, oxaliplatin, SN38) (n = 3 per group).

(E) EdU incorporation assay assessing proliferation in Capan-1 and MIA PaCa-2 cells with cCCT2 overexpression or knockdown (n = 6 per group). Scale bar, 50 µm.

(F) Western blot of apoptosis‑related proteins in MIA PaCa‑2 cells with sh‑NC or sh‑cCCT2.

(G) Western blot of senescence‑related proteins in MIA PaCa‑2 cells with sh‑NC or sh‑cCCT2.

The data are expressed as the means ± SDs. Unpaired two-tailed Student’s t-test or Mann-Whitney *U*-test (B up, D), one-way ANOVA with Tukey’s post-hoc test (B down), two-way ANOVA with Tukey’s post-hoc test (E). Not significant (ns); **P* < 0.05; ***P* < 0.01; ****P* < 0.001; *****P* < 0.0001.


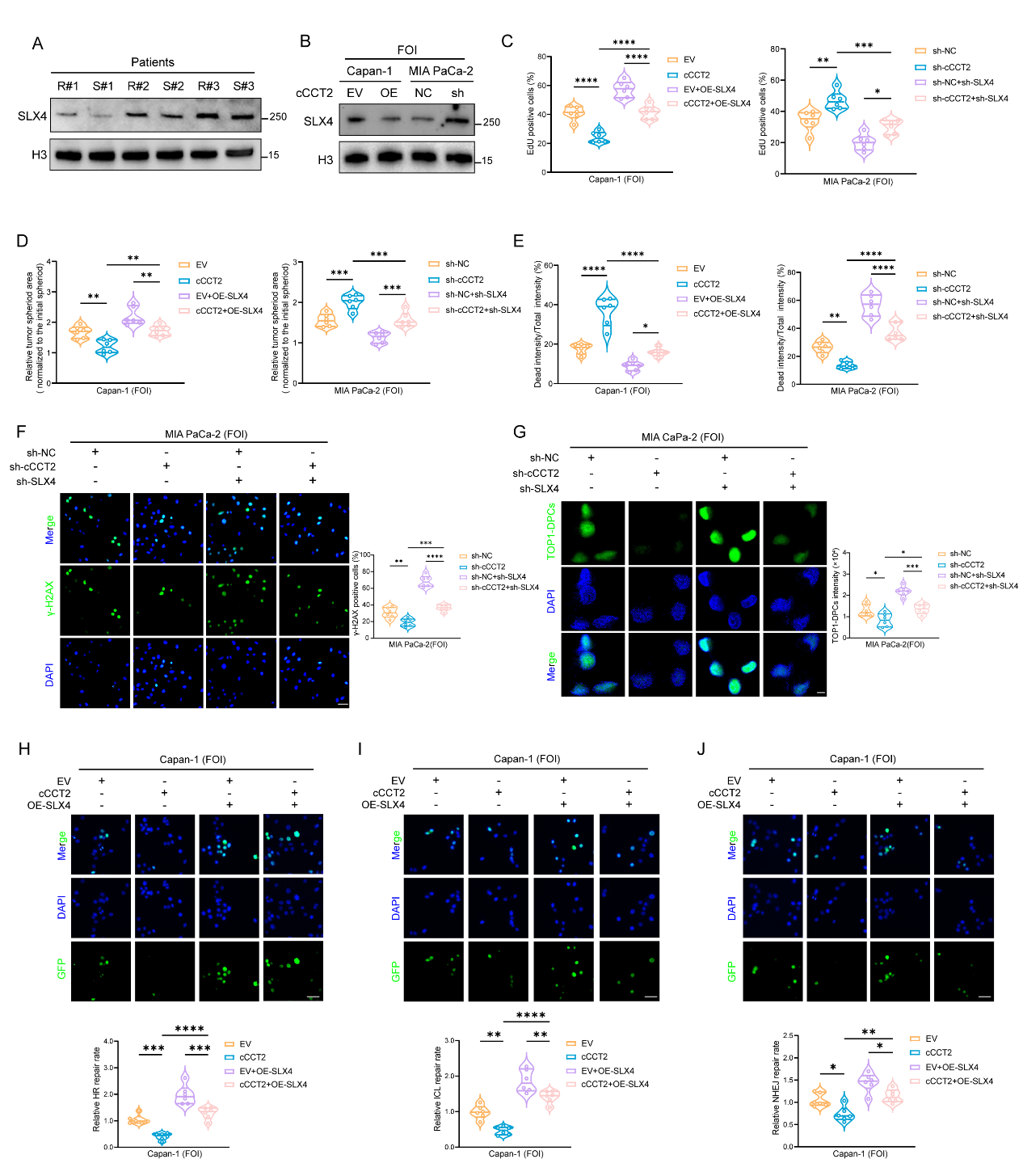


**Supplementary Figure S5**

(A) Western blot showing SLX4 expression in three pairs of chemotherapy resistant and sensitive patient samples.

(B) Western blot of SLX4 expression in Capan-1 and MIA PaCa-2 cells with cCCT2 overexpression or knockdown.

(C) EdU incorporation assay evaluating effects of altering SLX4 expression on proliferation of PDAC cells with different cCCT2 expression levels and quantification of the results (n = 6 per group).

(D) Assessment of the effect of altering SLX4 expression on proliferation of 3D microtumor spheroids with different cCCT2 expression levels (n = 6 per group).

(E) Assessment of the effect of altering SLX4 expression on apoptosis of 3D microtumor spheroids with different cCCT2 expression levels (n = 6 per group).

(F) Representative images of the effect of altering SLX4 expression levels on γ-H2AX accumulation in MIA PaCa-2 cells with sh-NC or knockdown cCCT2 and quantification of the results (n = 6 per group).

(G) Representative images of the effect of altering SLX4 expression levels on TOP1‑DPCs accumulation in MIA PaCa-2 cells with sh-NC or knockdown cCCT2 and quantification of the results (n = 6 per group). Scale bar, 20 µm.

(H) Representative images of the effect of altering SLX4 expression levels on HR repair rate in Capan-1 cells with EV or overexpressing cCCT2 and quantification of the results (n = 6 per group).

(I) Representative images of the effect of altering SLX4 expression levels on ICL repair rate in Capan-1 cells with EV or overexpressing cCCT2 and quantification of the results (n = 6 per group).

(J) Representative images of the effect of altering SLX4 expression levels on NHEJ repair rate in Capan-1 cells with EV or overexpressing cCCT2 and quantification of the results (n = 6 per group).

One-way ANOVA with Tukey’s post-hoc test (C-J). Not significant (ns); **P* < 0.05; ***P* < 0.01; ****P* < 0.001; *****P* < 0.0001.


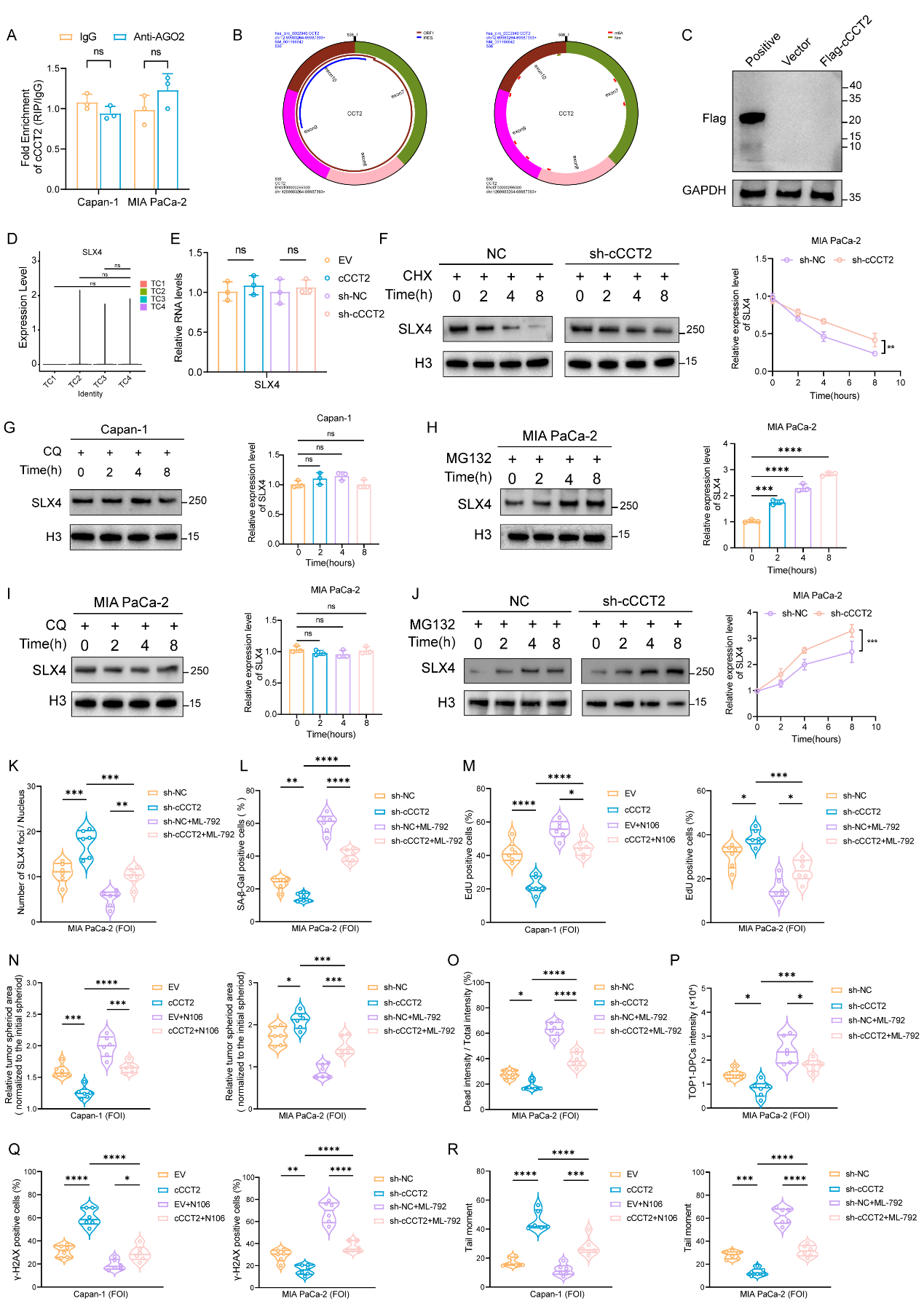


**Supplementary Figure S6**

(A) RT-qPCR was performed after the RIP experiments to detect the amount of cCCT2 bound to the AGO2 protein (n = 3 per group).

(B) Protein‑coding potential of cCCT2 predicted using circPrimer 2.0.

(C) Western blot detection of Flag‑tagged peptides encoded by cCCT2.

(D) Violin plots of SLX4 expression across tumor cell subpopulations in scRNA-seq data.

(E) RT-qPCR analysis of SLX4 mRNA levels at different cCCT2 expression levels (n = 3 per group).

(F) Degradation of the SLX4 protein in MIA PaCa-2 cells with sh-NC or knockdown cCCT2 after treatment with cycloheximide (CHX) for 2 h, 4 h, and 8 h and quantification of the results (n = 3 per group).

(G) Degradation of the SLX4 protein in Capan-1 cells treated with CQ for 2 h, 4 h, or 8 h and quantification of the results (n = 3 per group).

(H) Degradation of the SLX4 protein in MIA PaCa-2 cells treated with MG132 for 2 h, 4 h, or 8 h and quantification of the results (n = 3 per group).

(I) Degradation of the SLX4 protein in MIA PaCa-2 cells treated with CQ for 2 h, 4 h, or 8 h and quantification of the results (n = 3 per group).

(J) Degradation of the SLX4 protein in MIA PaCa-2 cells with sh-NC or knockdown cCCT2 after treatment with MG132 for 2 h, 4 h, and 8 h and quantification of the results (n = 3 per group).

(K) Assessment of the rescue effect of ML-792 on SLX4 condensate formation in MIA PaCa-2 cells with sh-NC or knockdown cCCT2 and quantification of the results (n = 6 per group).

(L) Assessment of the rescue effect of ML-792 on SA-β-Gal staining in MIA PaCa-2 cells with sh-NC or knockdown cCCT2 and quantification of the results (n = 6 per group).

(M) Assessment of the rescue effect of ML-792 on EdU incorporation in PDAC cells with different cCCT2 expression levels (n = 6 per group).

(N) Assessment of the rescue effect of N106 or ML-792 on proliferation of 3D microtumor spheroids with different cCCT2 expression levels (n = 6 per group).

(O) Assessment of the rescue effect of N106 or ML-792 on apoptosis of MIA PaCa-2 3D microtumor spheroids with sh-NC or knockdown cCCT2 (n = 6 per group).

(P) Assessment of the rescue effect of ML-792 on TOP1‑DPCs accumulation in MIA PaCa-2 cells with sh-NC or knockdown cCCT2 (n = 6 per group).

(Q) Assessment of the rescue effect of N106 or ML-792 on γ-H2AX accumulation in PDAC cells with different cCCT2 expression levels (n = 6 per group).

(R) Assessment of the rescue effect of N106 or ML-792 on tail moment of comet assay in PDAC cells with different cCCT2 expression levels (n = 6 per group).

Unpaired two-tailed Student’s t-test or Mann-Whitney *U*-test (A, E), one-way ANOVA with ​Dunnett's post-hoc test (D, G-I), repeated measures ANOVA test (F, J), one-way ANOVA with Tukey’s post-hoc test (K-R). Not significant (ns); **P* < 0.05; ***P* < 0.01; ****P* < 0.001; *****P* < 0.0001.

**
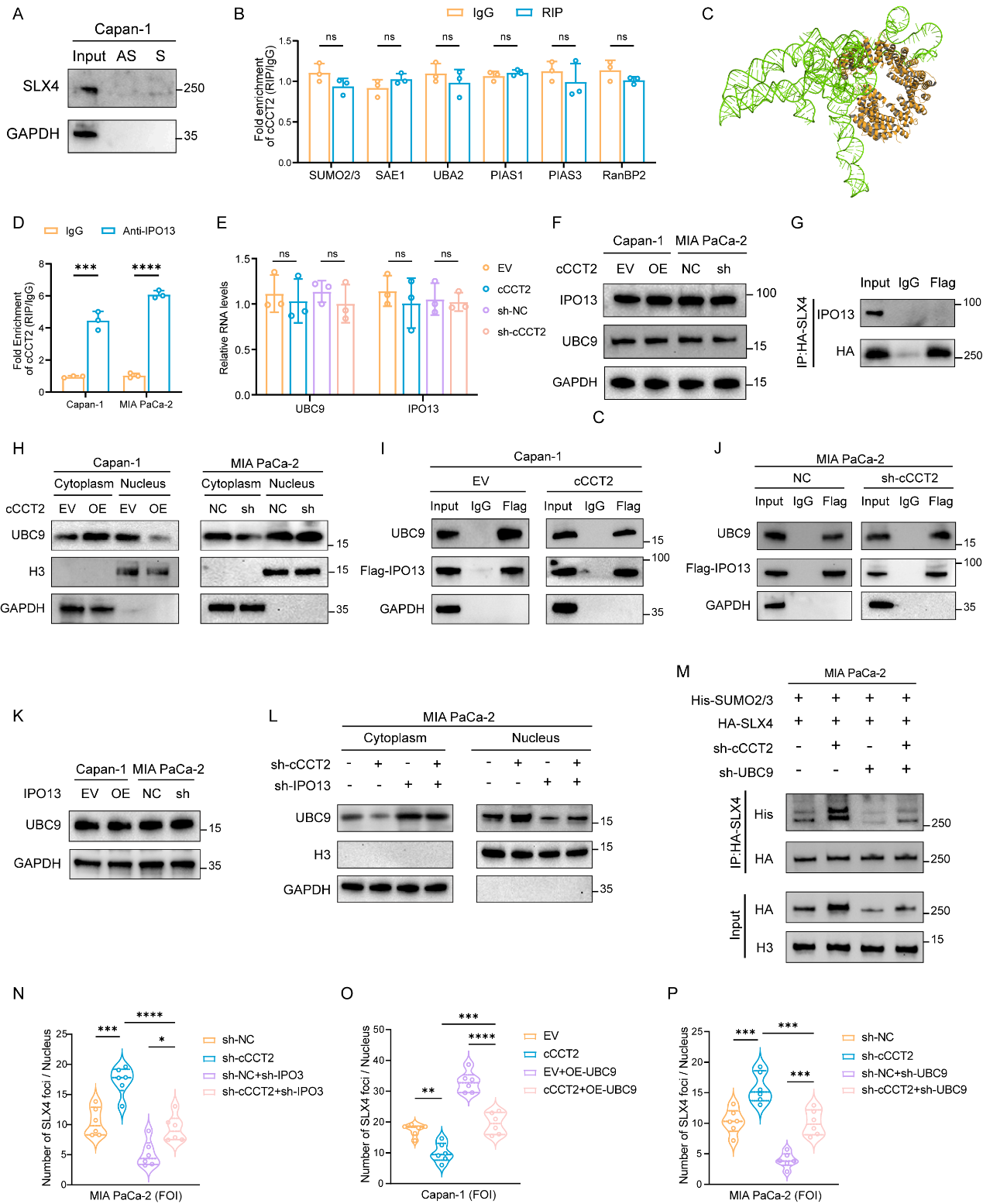
**

**Supplementary Figure S7**

(A) Interaction between cCCT2 and the SLX4 protein was detected in Capan-1 cells via a specific biotin-labelled cCCT2 (sense) RNA pull-down assay.

(B) RT-qPCR was performed after the RIP experiments to detect the amount of cCCT2 bound to the SUMOylation-related protein (n = 3).

(C) The interaction between the cCCT2 and IPO13 proteins was predicted using the HDOCK server. The cCCT2 secondary structure was predicted with the RNAfold web server according to the minimum free energy (MFE). The 3D structure of IPO13 was obtained from the PDB.

(D) RT-qPCR was performed after the CLIP experiments to detect the amount of cCCT2 bound to the IPO13 protein (n = 3).

(E) RT-qPCR analysis of UBC9 and IPO13 mRNA levels at different cCCT2 expression levels (n = 3 per group).

(F) Western blot analysis of UBC9 and IPO13 protein levels at different cCCT2 expression levels.

(G) Interaction between SLX4 and IPO13 was detected via the Co-IP assay.

(H) Assessment of nuclear and cytoplasmic distribution of UBC9 at different cCCT2 expression levels. H3 was used as a nuclear marker, and GAPDH was used as a cytoplasmic marker.

(I) Assessment of the interaction between IPO13 and UBC9 in Capan‑1 cells with EV or overexpressing cCCT2.

(J) Assessment of the interaction between IPO13 and UBC9 in MIA PaCa-2 cells with sh-NC or knockdown cCCT2.

(K) Western blot analysis of UBC9 protein levels at different IPO13 expression levels.

(L) Assessment of nuclear and cytoplasmic distribution of UBC9 in MIA PaCa-2 cells with sh-NC or knockdown cCCT2 following altering IPO13 expression levels. H3 was used as a nuclear marker, and GAPDH was used as a cytoplasmic marker.

(M) Measurement of SLX4 protein expression and SUMOylation levels in Capan‑1 cells with sh-NC or knockdown cCCT2 following the knockdown of UBC9.

(N) Assessment of the effect of altering IPO13 expression levels on SLX4 condensation formation in MIA PaCa-2 cells with sh-NC or knockdown cCCT2 (n = 6 per group).

(O) Assessment of the effect of altering UBC9 expression levels on SLX4 condensation formation in Capan‑1 cells with EV or overexpressing cCCT2 (n = 6 per group).

(P) Assessment of the effect of altering UBC9 expression levels on SLX4 condensation formation in MIA PaCa-2 cells with sh-NC or knockdown cCCT2 (n = 6 per group).

Unpaired two-tailed Student’s t-test or Mann-Whitney *U*-test (B, D, E), one-way ANOVA with Tukey’s post-hoc test (N-P). Not significant (ns); **P* < 0.05; ***P* < 0.01; ****P* < 0.001; *****P* < 0.0001.

**
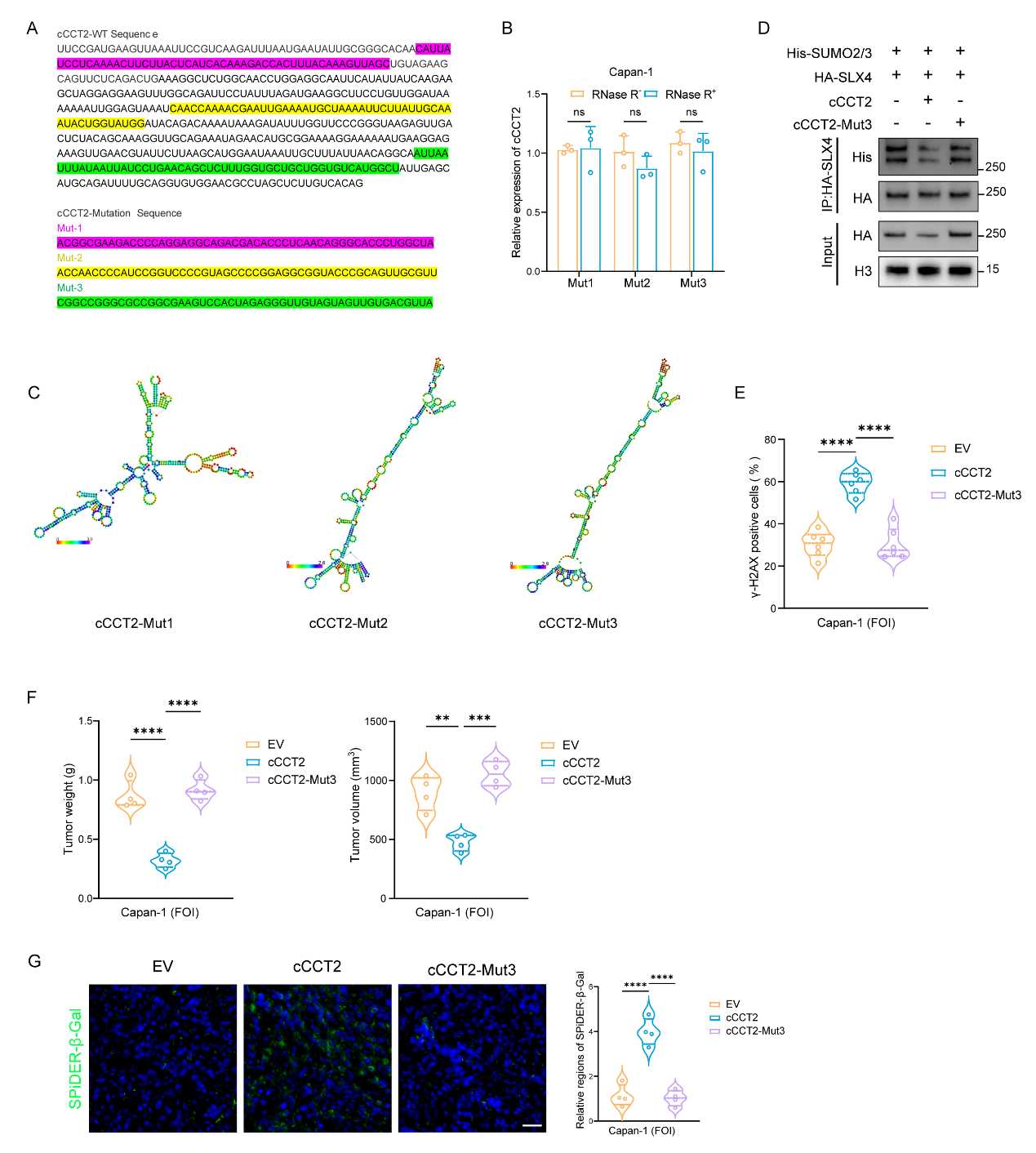
Supplementary Figure S8**

(A) Sequences of c cCCT2 Mut 1 (51-102 nt), Mut 2 (236-287 nt), and Mut 3 (436-487 nt).

(B)  RNA stability analysis of cCCT2 Mut 1, Mut 2, and Mut 3 in RNase R-treated Capan-1 cells (n = 3 per group).

(C) Prediction of stem-loop structural regions on cCCT2 Mut 1, Mut 2, and Mut 3 via RNAfold server.

(D) Measurement of SLX4 protein expression and SUMOylation levels in Capan‑1 cells with cCCT2-Mut 3.

(E) Assessment of the effect of cCCT2-Mut 3 on γ-H2AX accumulation in Capan-1 cells (n = 6 per group).

(F) Tumor volume and tumor weight for xenograft mouse models generated with cCCT2-overexpressing or cCCT2-Mut3 Capan-1 cells (n = 4 per group).

(G) Representative senescence-associated fluorescent staining images of in situ pancreatic tumors with cCCT2-overexpressing or cCCT2-Mut3 and quantification of the results (n = 4 per group). Scale bar, 100 µm.

Unpaired two-tailed Student’s t-test or Mann-Whitney *U*-test (B), one-way ANOVA with ​Dunnett's post-hoc test (E-G). Not significant (ns); **P* < 0.05; ***P* < 0.01; ****P* < 0.001; *****P* < 0.0001.

**
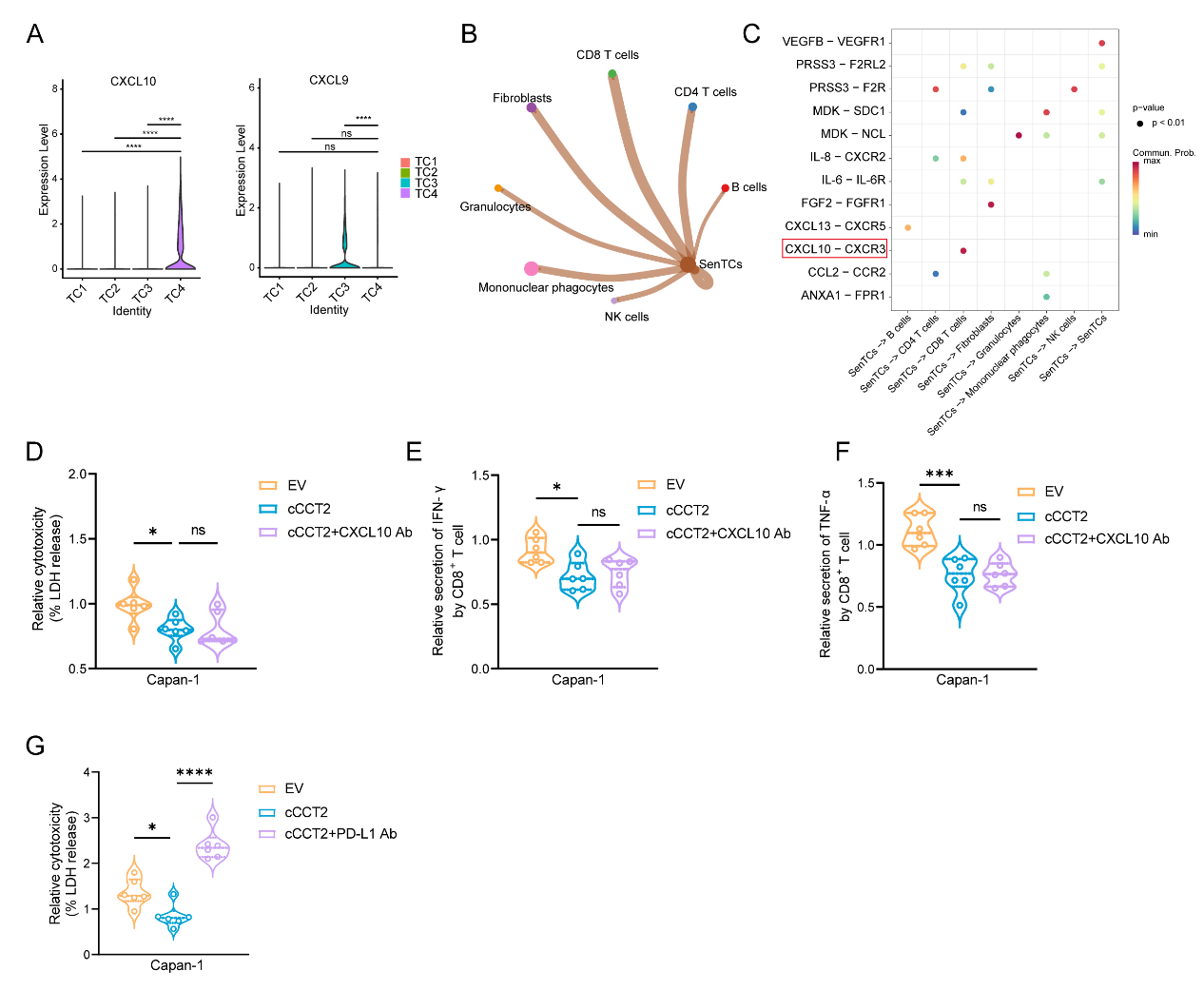
**

**Supplementary Figure S9**

(A) Violin plots of CXCL10 and CXCL9 expression across tumor cell subpopulations in scRNA-seq data.

(B) Interaction network displays communication between senescent tumor cells (senTCs) and immune cells in scRNA-seq data.

(C) Communication probabilities of key ligand-receptor pairs between senTCs and immune cells in scRNA-seq data.

(D) Cytotoxicity mediated by CD8^+^ T-cells against Capan-1 cells with EV or overexpressing cCCT2 under CXCL10 neutralising antibody, as measured by lactate dehydrogenase (LDH) release (n = 6 per group).

(E) ELISA quantification of IFN-γ levels in the culture supernatants of CD8⁺ T cells co‑cultured with Capan‑1 cells with EV or overexpressing cCCT2 under CXCL10 neutralising antibody blockade (n = 6 per group).

(F) ELISA quantification of TNF-α levels in the culture supernatants of CD8⁺ T cells co‑cultured with Capan‑1 cells with EV or overexpressing cCCT2 under CXCL10 neutralising antibody blockade (n = 6 per group).

(G) Cytotoxicity mediated by CD8^+^ T-cells against Capan-1 cells with EV or overexpressing cCCT2 under anti-PD-L1 antibody blockade, as measured by LDH release (n = 6 per group).

One-way ANOVA with ​Dunnett's post-hoc test (A), one-way ANOVA with Tukey’s post-hoc test (D-G). Not significant (ns); **P* < 0.05; ***P* < 0.01; ****P* < 0.001; *****P* < 0.0001.


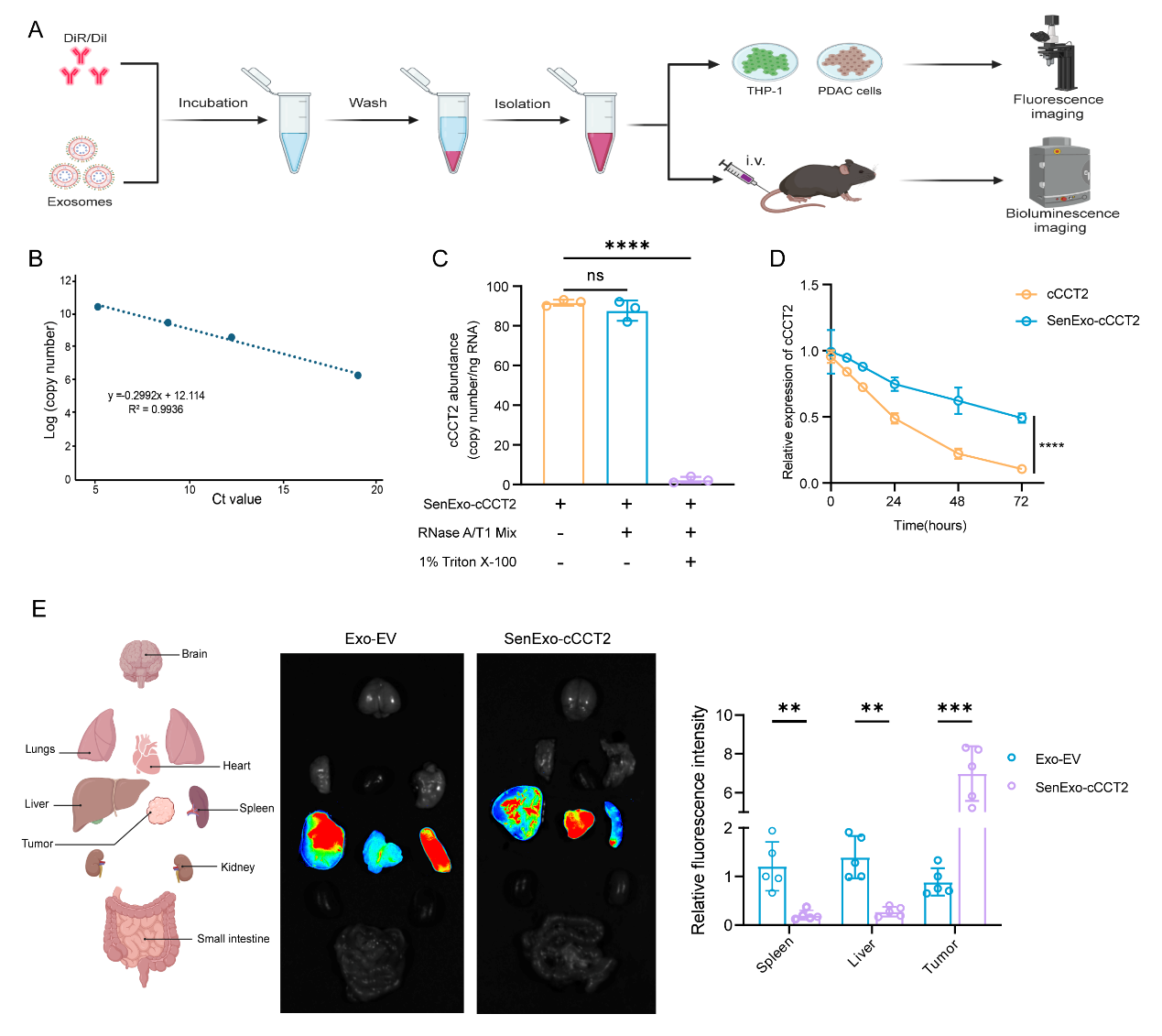


**Supplementary Figure S10**

(A) Schematic diagram of DiR‑ or DiI‑labelled SenExo‑cCCT2 targeting to tumor cells and in vivo organ distribution.

(B) The standard curves of log cCCT2 copy numbers and the Ct values as determined by qRT-PCR.

(C) Absolute qPCR analysis of cCCT2 copy numbers in SenExo‑cCCT2 after treatments with RNase A/T1 Mix and 1% Triton X‐100 for 30 min (n = 3 per group).

(D) RT-PCR analysis of cCCT2 abundance in serum collected at various time points after injection of naked cCCT2 versus SenExo-cCCT2 (n = 3 per group).

(E) Ex vivo fluorescence images of tumor and major organs following DiR‑labelled senExo‑cCCT2 injection and quantification of the results (n = 5 per group).

Unpaired two-tailed Student’s t-test or Mann-Whitney *U*-test (E), one-way ANOVA with ​Dunnett's post-hoc test (C), repeated measures ANOVA test (D). Not significant (ns); **P* < 0.05; ***P* < 0.01; ****P* < 0.001; *****P* < 0.0001.

**
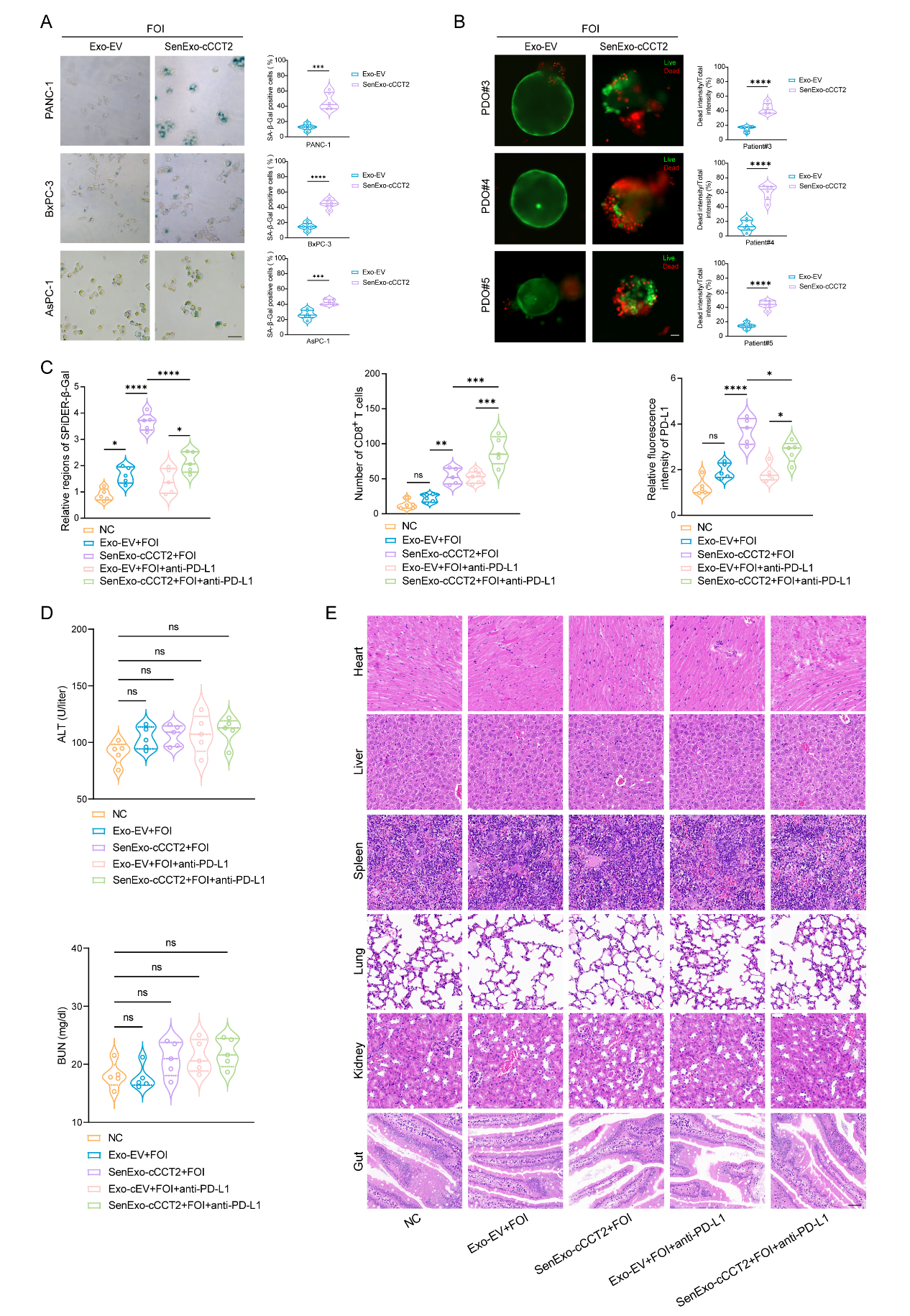
**

**Supplementary Figure S11**

(A) SA-β-Gal staining of PDAC cells harboring different mutations after SenExo-cCCT2 intervention and quantification of the results (n = 6 per group). Scale bar, 30 µm.

(B) Assessment of the effect of SenExo-cCCT2 intervention on the apoptosis of patient-derived organoids (PDO) and quantification of the results (n = 6 per group). Live cells were stained with calcein-AM (green), and dead cells were stained with PI (red). Scale bar, 50 µm.

(C) Quantification of SPiDER-β-Gal, CD8, and PD-L1 expression in tumor mIF images (n = 5 per group).

(D) Serum alanine aminotransferase (ALT) and urea nitrogen (BUN) levels measured after the third treatment cycle (n = 5 per group).

(E) Representative H&E‑stained images of major organs from Hu-NSG bearing PDX models treated with different combinations of SenExo-cCCT2, FOI, and anti-PD-L1 antibodies. Scale bar, 50 µm.

Unpaired two-tailed Student’s t-test or Mann-Whitney *U*-test (A, B), one-way ANOVA with ​Dunnett's post-hoc test (D), one-way ANOVA with Tukey’s post-hoc test (C).Not significant (ns); **P* < 0.05; ***P* < 0.01; ****P* < 0.001; *****P* < 0.0001.

**
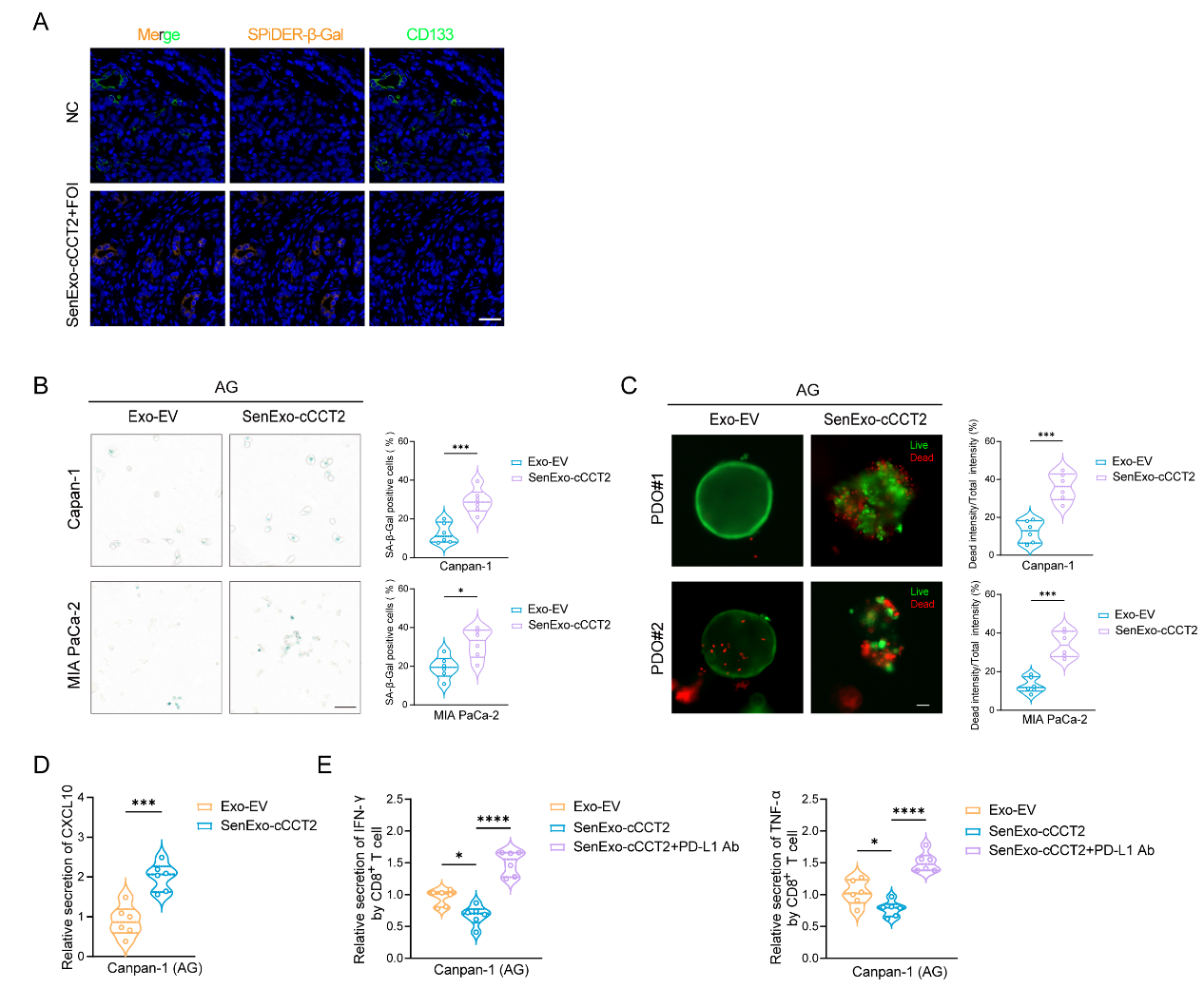
**

**Supplementary Figure S12**

(A) Representative mIF images of senescence (SPiDER-β-Gal) and stemness (CD133) markers in tumors from Hu-NSG bearing PDX models after treatment of SenExo-cCCT2 and FOI. Scale bar, 100 µm.

(B) SA-β-Gal staining of PDAC cells after SenExo-cCCT2 and AG intervention and quantification of the results (n = 6 per group). Scale bar, 30 µm.

(C) Assessment of the effect of SenExo-cCCT2 and AG intervention on the apoptosis of patient-derived organoids (PDO) and quantification of the results (n = 6 per group). Live cells were stained with calcein-AM (green), and dead cells were stained with PI (red). Scale bar, 50 µm.

(D) ELISAs of CXCL10 levels in Capan-1 cells after treatment of SenExo-cCCT2 and AG. (n = 6 per group).

(E) ELISAs of IFN-γ and TNF-α levels in the culture supernatants of CD8⁺ T-cells co‑cultured with SenExo-cCCT2/AG-induced senescent tumor cells (senTCs) under anti-PD-L1 antibody blockade (n = 6 per group).

Unpaired two-tailed Student’s t-test or Mann-Whitney *U*-test (B-D), one-way ANOVA with ​Dunnett's post-hoc test (E). Not significant (ns); **P* < 0.05; ***P* < 0.01; ****P* < 0.001; *****P* < 0.0001.

**Supplementary Tables**

**Supplementary table S1. Clinopathologic baseline characteristics of PDAC patients for single-cell RNA sequencing**

| **Sample ID** | **Gender** | **Age** | **Chemotherapy schedule** | **Response** | **Site of lesion** | **Tissue** | **T** | **N** | **M** | **Kras mutation** | **TP53 mutation** |
| --- | --- | --- | --- | --- | --- | --- | --- | --- | --- | --- | --- |
| CA01 | M | 70 | AG | Resistant | Body | Tumor Resection (fresh) | T3 | N1 | M0 | G12R | Mutation |
| CA02 | F | 52 | FOLFIRINOX | Sensitive | Body | Tumor Resection (fresh) | T2 | N2 | M0 | G12D | Mutation |
| CA03 | M | 58 | AG | Sensitive | Head | Tumor Resection (fresh) | T2 | N1 | M0 | G12D | WT |
| CA04 | M | 57 | FOLFIRINOX | Resistant | Head | Tumor Resection (fresh) | T2 | N1 | M0 | G12D | WT |
| CA05 | F | 53 | AG | Resistant | Head | Tumor Resection (fresh) | T2 | N2 | M0 | G12D | Mutation |
| CA06 | M | 67 | FOLFIRINOX | Sensitive | Head | Tumor Resection (fresh) | T3 | N1 | M0 | G12R | Mutation |

F, female; M, male; WT, wild type

**Supplementary table S2. Clinopathologic baseline characteristics of PDAC patients in this study**

| **Characteristics** | **Low cCCT2-expression** | **High cCCT2-expression** | **P value** |
| --- | --- | --- | --- |
| n | 50 | 46 |  |
| **Age** |  |  | 0.503 |
| ＜65 | 26 | 28 |  |
| ≥65 | 24 | 18 |  |
| **Gender** |  |  | 0.349 |
| Female | 22 | 15 |  |
| Male | 28 | 31 |  |
| **CA 19-9** |  |  | 0.606 |
| ＜100U/m | 21 | 16 |  |
| ≥ 100U/m | 29 | 30 |  |
| **T** |  |  | 0.006 |
| T2 | 0 | 1 |  |
| T3 | 28 | 38 |  |
| T4 | 22 | 7 |  |
| **N** |  |  | 0.254 |
| N0 | 20 | 26 |  |
| N1 | 26 | 18 |  |
| N2 | 4 | 2 |  |
| **M** |  |  | 0.342 |
| M0 | 40 | 41 |  |
| M1 | 10 | 5 |  |
| **TNM** |  |  | 0.014 |
| IB | 0 | 1 |  |
| IIA | 10 | 22 |  |
| IIB | 11 | 11 |  |
| III | 19 | 7 |  |
| IV | 10 | 5 |  |
| **KRAS mutation** |  |  | 0.861 |
| G12D | 21 | 18 |  |
| G12R | 5 | 6 |  |
| G12V | 15 | 13 |  |
| Other | 2 | 4 |  |
| WT | 7 | 5 |  |
| **TP53 mutation** |  |  | 0.798 |
| Mutation | 37 | 32 |  |
| WT | 13 | 14 |  |
| **PD-L1 expression** |  |  | 0.001 |
| Negative | 40 | 21 |  |
| Positive | 10 | 25 |  |

WT, wild type

**Supplementary Table S3. Primers and RNA sequences used in this study**

| **List of oligonucleotide sequences** | **5'→ 3'** |
| --- | --- |
| **Primers for RT-qPCR** |  |
| hsa_circ_0002940-F | GCTCTTGTCACAGTTCCGATG |
| hsa_circ_0002940-R | GGTTGCCAGAGCCTTTCAGT |
| CCT2-F(homo） | GTGTGGAACGCCTAGCTCTT |
| CCT2-R(homo） | TGGTACAAGCCTCACCAAGG |
| GAPDH-F(homo） | GGTGTGAACCATGAGAAGTATGA |
| GAPDH-R(homo） | GAGTCCTTCCACGATACCAAAG |
| U6-F(homo） | CTCGCTTCGGCAGCACA |
| U6-R(homo） | AACGCTTCACGAATTTGCGT |
| SLX4-F(homo) | GTGCTGAAGAAGGAACTGGATAG |
| SLX4-R(homo) | CTTGAAGGCTTGTAGGTCTGG |
| SAE1-F(homo) | TGCCAAAGTTAGCCAAGGAGT |
| SAE1-R(homo) | CAGGGCAGAAGACCACCTTC |
| UBA2-F(homo) | GTAAGCAACCTCAACAGACAGT |
| UBA2-R(homo) | TCATGGTAGGCAACGATATTAGC |
| UBC9-F(homo) | GGAGGCCAGCCATCACAATC |
| UBC9-R(homo) | GGCGCAAACTTCTTGGCTTG |
| IPO13-F(homo) | TCAGACGAGAAGGAGCAGTT |
| IPO13-R(homo) | GATGGATTGGAAGCCGTAGAG |
| PD-L1-F(homo） | AGGCCGAAGTCATCTGGACA |
| PD-L1-R(homo） | TGTTGATTCTCAGTGTGCTGGT |
| PD-L2-F(homo） | TACATCCCCACACCGTGAAAG |
| PD-L2-R(homo） | GGTACTGTCCTTCGTCCCTC |
| B7-H3-F(homo) | GGCTGTCTGTCTGTCTCATTG |
| B7-H3-R(homo) | CAGGCTATTTCTTGTCCATCATCT |
| Vista-F(homo） | TGCGGATGGACAGCAACATT |
| Vista-R(homo） | CTGGGCTCCGAAAGCAGATG |
| TIM-3-F(homo) | GATGCTTACCACCAGGGGAC |
| TIM-3-R(homo) | CACAGATCCCTGCTCCGATG |
| LAG-3-F(homo) | GGCGACTTTACCCTTCGACT |
| LAG-3-R(homo) | ATGTGACAGTGGCATTGAGC |
| CD155-F(homo) | TGGAGCACGAGAGCTTTGAG |
| CD155-R(homo) | GCCTCATTCTGGCCAAGGTA |
| GAL-9-F(homo) | CAGACTGGCTTCAGTGGAAATGA |
| GAL-9-R(homo) | CCTTCTGGAAAGGCATGTGTG |
| **shRNAs** |  |
| hsa_circ_0002940-shRNA#1 | GTCACAGTTCCGATGAAGT |
| hsa_circ_0002940-shRNA#2 | GCTCTTGTCACAGTTCCGA |
| Homo-SLX4-shRNA | GCACTCAGATAAGAAGCAAATTGAA |
| Homo-IPO13-shRNA | CAGCTCTACTATGATCCCAACATTG |
| Homo-UBC9-shRNA | AAGCATGGAGGAAAGACCACCCATT |

F, forward; R, reverse.
